# Bereavement care interventions: a systematic review

**DOI:** 10.1186/1472-684X-3-3

**Published:** 2004-07-26

**Authors:** Amanda L Forte, Malinda Hill, Rachel Pazder, Chris Feudtner

**Affiliations:** 1 Pediatric Advanced Care Team and Pediatric Generalist Research Group, Division of General Pediatrics, The Children's Hospital of Philadelphia, PA, USA; 2 Department of Social Work and Family Services, The Children's Hospital of Philadelphia, PA, USA; 3 The Leonard Davis Institute of Health Economics, University of Pennsylvania, Philadelphia, PA, USA; 4 Center for Bioethics, University of Pennsylvania, Philadelphia, PA, USA

**Keywords:** Bereavement, intervention, systematic review

## Abstract

**Background:**

Despite abundant bereavement care options, consensus is lacking regarding optimal care for bereaved persons.

**Methods:**

We conducted a systematic review, searching MEDLINE, PsychINFO, CINAHL, EBMR, and other databases using the terms (bereaved or bereavement) and (grief) combined with (intervention or support or counselling or therapy) and (controlled or trial or design). We also searched citations in published reports for additional pertinent studies. Eligible studies had to evaluate whether the treatment of bereaved individuals reduced bereavement-related symptoms. Data from the studies was abstracted independently by two reviewers.

**Results:**

74 eligible studies evaluated diverse treatments designed to ameliorate a variety of outcomes associated with bereavement. Among studies utilizing a structured therapeutic relationship, eight featured pharmacotherapy (4 included an untreated control group), 39 featured support groups or counselling (23 included a control group), and 25 studies featured cognitive-behavioural, psychodynamic, psychoanalytical, or interpersonal therapies (17 included a control group). Seven studies employed systems-oriented interventions (all had control groups). Other than efficacy for pharmacological treatment of bereavement-related depression, we could identify no consistent pattern of treatment benefit among the other forms of interventions.

**Conclusions:**

Due to a paucity of reports on controlled clinical trails, no rigorous evidence-based recommendation regarding the treatment of bereaved persons is currently possible except for the pharmacologic treatment of depression. We postulate the following five factors as impeding scientific progress regarding bereavement care interventions: 1) excessive theoretical heterogeneity, 2) stultifying between-study variation, 3) inadequate reporting of intervention procedures, 4) few published replication studies, and 5) methodological flaws of study design.

## Background

Give sorrow words; the grief that does not speak

Whispers the o'er fraught heart and bids it break.

Shakespeare, *Macbeth *IV, iii, 209

Grieving the death of a loved one has an ancient history: from time immemorial, cultures have provided the bereaved with advice and rituals to address – and express – the experience of grief [1]. Over the past several decades, efforts to aid the bereaved have increasingly focused on the physical and psychological morbidity, and the spiritual suffering and social isolation associated with bereavement. The resulting plethora of intervention options, ranging from mutual-help support groups to prescribed pharmacotherapy and professionally led psychotherapy, is striking, as is the panoply of settings in which bereavement care can be found: hospitals, hospices, churches, palliative care units, community-based services, and bereavement-specific foundations all provide an array of bereavement care interventions. This welter of activity testifies to the broadly valued goal of decreasing the severity of bereavement-related symptoms.

Given the abundance of care options, what is the best way to care for a bereaved person? Numerous studies measuring the impact of bereavement interventions have been published in diverse journals, yet no consensus has emerged in the medical, mental health, or social work communities regarding whether one form of treatment is preferable to another [2-5]. We therefore have conducted a systematic review of bereavement care interventions. Our goal is to present a comprehensive yet coherent synthesis of the current literature that will promote the advancement in the quality of care and research on behalf of bereaved individuals.

## Methods

### Data sources

To identify studies in the traditional medical literature as well as the complementary and alternative medicine literature, we searched the following databases: MEDLINE; PsychINFO; Cumulative Index to Nursing and Allied Health (CINAHL); BIOSIS Previews; ISI Science Citation Index Expanded and Social Sciences Index; Evidence Based Medicine Reviews (EBMR), including the Cochrane Database of Systematic Reviews (DSR), the Cochrane Controlled Trial Registry (CCTR), Database of Abstracts of Reviews of Effectiveness (DARE), and the American College of Physicians' (ACP) Journal Club Review; Sociological Abstracts; Alt HealthWatch; and Wilson Web from 1966 to 2003. We identified all relevant articles on bereavement care interventions by using the primary search terms of "*bereaved *or *bereavement*" and "*grief*", combined with secondary descriptors of "*intervention *or *support *or *counselling *or *therapy*" and "*controlled *or *trial *or *design*".

### Study selection

Our inclusion criteria specified that each study: 1) addressed the treatment of bereaved individuals, and 2) included an evaluation of a selected method of therapy aimed at reducing the grief reaction due to bereavement. We considered only articles written in the English language. We then reviewed the titles and abstracts of all articles we retrieved through our initial database search, and obtained the full texts of all applicable studies. We also reviewed the references in all applicable studies for additional pertinent studies.

### Data extraction

The full articles of all studies that met inclusion criteria and passed subsequent title and abstract reviews were retrieved and examined independently by two of the authors. Each article was reviewed for measured outcomes, patient and decedent characteristics, and intervention characteristics. These measures included sample size, type of intervention, length of intervention, patient's relationship to the deceased, time since the bereaved death, and patient demographics. Data was extracted and any disagreements were resolved through discussion, clarification, and consensus within the research team.

### Characteristics of reviewed studies

The initial literature search generated 737 citations. Elimination of duplicate citations yielded 340 references. 2 studies, written in Chinese and Spanish, were excluded. Reviewing the titles culled the sample to 243 citations, and a review of the abstracts found 87 of these to be potentially relevant. Of these, 9 were dissertations, 2 were irretrievable, 2 were duplicate publications of the same study, and 15 were ineligible because they did not meet our inclusion criteria. The resulting set of 74 articles was subject to review for data extraction. A list of all citations found, including those excluded from this analysis, is available [see Additional file 1].

Of the 74 studies that met inclusion criteria, almost 6,000 participants within these studies experienced a multitude of losses – of parents, spouses, children, and other loved ones who had died from a wide range of causes, both sudden and protracted. The therapies utilized and outcomes evaluated varied widely. Heterogeneity among both the outcomes and the measures used to assess similar outcomes precluded an effort to summarize data across studies, even in the form of generic effect-size measures. Furthermore, for a significant portion of the studies, concerns regarding the internal or external validity of the reported results cautioned against making quantitative summary statements regarding treatment effects.

## Results

The 74 studies selected for detailed review evaluated diverse types of interventions designed to ameliorate the adverse physical and psychological outcomes associated with bereavement. These interventions can be classified according to various schemes, including their underlying theoretical framework (ranging from Freudian psychoanalysis to neurotransmitter imbalances), the format of the intervention (individual, group, family, marital), the timing of the intervention (acute, intermittent crisis, chronic), the tasks assigned to the bereaved (ranging from verbalizing feelings to taking medication), or the population targeted for the intervention (children, adults, seniors). We chose to organize this review on the basis of the social framework used to implement the intervention (that is, either personalized structured therapeutic relationships or less personal systems-level interventions), as this attribute of the interventions emerged as the most verifiable and salient measure.

### Structured therapeutic relationship

Eight studies feature ***pharmacotherapy***, but only four compared active therapy to non-pharmacotherapy controls, and only one study clearly reported their random allocation method (Table 1) [6-13]. These studies targeted adults and seniors, ranged in sample size from 10–80 subjects, and used a variety of drugs, including tricyclic antidepressants (TCA), selective serotonin reuptake inhibitors (SSRI), buprioion, and benzodiazepines. Overall, these studies demonstrated a statistically significant beneficial effect of pharmacotherapy on ameliorating symptoms of depression and improving subjective sleep quality [6-11,13]. These benefits persisted only as long as the subjects continued to receive pharmacotherapy. Pharmacotherapy was found, however, to have a mixed effect on bereavement intensity as measured by symptoms of grief (i.e., Texas Revised Inventory of Grief, Inventory of Complicated Grief). For example, Warner and colleagues (2001) did not find evidence of an effect of benzodiazepines (diazepam) on bereavement-related grief intensity[12]. One study combined pharmacotherapy with psychotherapy in a 16-week double-blinded factorial design trial of nortriptyline (NT) and interpersonal psychotherapy [6]. The 80 patients were randomly assigned to one of four treatment conditions: NT plus interpersonal psychotherapy, NT plus medication clinic (i.e., no interpersonal psychotherapy), placebo pill plus interpersonal psychotherapy, and placebo pill plus medication clinic (i.e., no interpersonal psychotherapy conditions). Details of the psychotherapy were not described. While the results displayed a statistically significant benefit of nortriptyline over placebo regarding remission of depression, none of the treatment conditions were associated with diminishment of grief.

**Table 1:** Pharmacotherapy Interventions

**Medication**	**Pop**	**CG**	**RA**	Num*	**TSL (days)**	**Dose**	**DT (days)**	**Key Outcome Measures**	**Article**
Nortriptyline	Senior	Y	Y-NE	80/66	216–279	Steady-state plasma level: 50–120 ng/mL	112	Depression (HAM-D); Grief (TRIG)	Reynolds, Miller, et al, 1999**
	Senior	Y	Y-NE	27/27	210 (mean)	Steady-state plasma level: 79.9+/-28.3 ng/mL Daily dose: 70.8+/-22.2 mg	<180	Sleep (PSQI); Depression (HAM-D, BDI)	Taylor, Reynolds, et al, 1999
	Senior	Y	NR	30/24	276	Steady-state plasma level: 72.7 ng/mL Daily dose: 53.0 mg	112	Sleep (PSQI)	Pasternak, Reynolds, et al, 1994
	Senior	N	NA	13/13	150–750	Daily dose: 49.2 mg	9–184	Depression (HAM-D, BDI, BSI); Grief (TRIG, JGI); Sleep (PSQI)	Pasternak, Reynolds, et al, 1991
Nortriptyline and Paroxetine	Adult	N	NA	21/15	183–4158	PT Daily dose: 20–50 mg NT Daily dose: 50–160 mg	120	Depression (HAM-D); Grief (ICG); Sleep (PSQI)	Zygmont, Prigerson, et al, 1998
Desipramine	Adult	N	NA	10/9	NR	Daily dose: 75–150 mg	28	Depression (HDRS, CGI, Raskin DS); Grief (Separation Distress)	Jacobs, Nelson, et al, 1987
Bupropion	Adult	N	NA	22/14	42–56	Daily dose: 150–300 mg	56	Grief (TRIG, ICG); Depression (HAM-D)	Zisook, Schuchter, et al, 2001
Diazepam	Senior	Y	Y	35/30	<14	2 mg/pill, self-administered	<42	Bereavement (BPQ)	Warner, Metcalfe, et al, 2001

Notes: * All Ns are reported as (starting population of bereaved individuals/bereaved population completing all follow-ups), unless only study included only one assessment. ** Study also included psychotherapy condition. Legend: Pop, Target Population; CG, Control Group; RA, Random Assignment; Num, Number of subjects; TSL, Time Since Loss; DT, Duration of Trial; NA, Not Applicable; NR, Not Reported; UC, Unclear; Y, Yes; N, No; Y-NE, Randomization mentioned, but allocation method not explicitly stated; RS, Randomization Subverted.

***Support groups or counselling ***constituted the intervention in 39 studies, of which 23 had control groups and 15 claimed random allocation, yet only three of these included clearly described allocation methods (Table 2) [14-52]. Ten of these were mutual/self-help, with the majority taking the form of informal group therapy. The remaining 29 studies were professionally led support groups targeting select subgroups including parentally bereaved children, college students, and seniors, as well as many specific adult populations. Program implementation across studies varied even further. This variation was found in terms of number of sessions (one to 25), whether the sessions proceeded with full-fledged patient-driven discussion or highly structured protocols, whether attendance was mandatory or individually motivated, as well as in the nature of the group leadership and the format (individual, group, or marital). Perhaps due to these or other differences in the interventions, some studies documented study treatment effects [22,26,29-31,33,34,52] while other studies showed no effect [15,17,27,37,46,51].

**Table 2:** Support/Counselling Interventions

**Type**	**Format**	**Pop**	**CG**	**RA**	**Num**	**TSL (days)**	**DT**	**Key Outcome Measures**	**Article**
Mutual/Self-help	Individual	Adult	Y	Y-NE	162/62	~30	NR	Psychiatric Functioning (GHQ); Social Support/psychological and psychophysiological variables (author-created)	Vachon, Lyall, et al, 1980
Mutual/Self-help (included professionally-lead groups)	Group	Senior	Y	RS	339/295	30–60	56, 365 days	Self-Esteem (Rosenberg's Self-Esteem Scale); Life Satisfaction (LSI-A); Depression (GDS); Grief (TRIG)	Caserta & Lund, 1983
Mutual/Self-help	Group	Senior	Y	N	23	34–474	21 days; 7 sessions	Domain Specific State Locus of Control (Zeigler-Reid State Locus of Control Measure); Trait Locus of Control (I-E); Distress (BSI, GSI)	McKibbin, Guarnaccia, et al, 1997
Mutual/Self-Help	Group	Adult	Y	Y	113/67	90–365	63 days; 9 sessions	Depression (GHQ, BDI); Anxiety (STAI); Social Functioning (SAS); Social Support (SSQ)	Tudiver, Hilditch, et al, 1992
Mutual/Self-help	Group	Adult	Y	Y-NE	113/112	90–365	63 days	Healthcare visit rates (Family Physician, Specialist, Psychiatrist)	Tudiver, Permaul-Woods, et al, 1995
Mutual/Self-help	Group	Adult	Y	N	38/21	90–750	70 days; 10 sessions	Treatment Expectancy (Expectancy Scale); Depression (BDI); Avoidance, Anxiety (Social Anxiety and Distress Scale); Enjoyability (Pleasant Events Scale); Life Satisfaction (Life Satisfaction Scale)	Walls & Meyers, 1985
Mutual/Self-help	Group	Adult	Y	N	721/502	~1290	365 days; >3 sessions	Depression, Anxiety, Somatization (Hopkins Symptom Checklist); Self Esteem, Well-being, Mastery (Not reported)	Lieberman & Videka-Sherman, 1986
Mutual/Self-help	Group	Adult	Y	N	667/391	365–1095	365 days	Depression, Anxiety, Somatization (Not reported); Self Esteem (Rosenberg 1965); Life Satisfaction, Mastery, Medication (Not reported); Social Functioning Parental Functioning Attitudes (BPQ)	Videka-Sherman & Lieberman, 1985
Mutual/Self-help	Group	Adult	N	Y-NE	61/55	120–1095	84 days; 12 sessions	Avoidance/Intrusion (IES); Stress Symptoms (SRRS); Depression (BDI); Mental Distress (BPRS, SCL-90); Social Functioning (SAS-SR); Overall Functioning (GAS)	Marmar, Horowitz, et al, 1988**
Mutual/Self-help	Group	Adult	N	NA	53/33	<730	8 sessions, optional 4	Psychosomatic Symptoms (SCL-90 subscales: somatization, obsessive-compulsive, interpersonal sensitivity, depression, anxiety, hostility, phobic anxiety, paranoid ideation, psychoticism, GSI)	Rogers, Sheldon, et al, 1982
Professionally Lead	Individual	Adult	Y	NR	493/225	120	1 day; 1 session	Grief (HGRC)	Kaunonen, Tarkka, et al, 2000
Professionally Lead	Family	Adult	Y	Y-NE	50/30	1–2	1–120 days; up to 8 sessions	General Health Questionnaire (self-rated); Anxiety, Depression (Leeds Scale)	Forrest, Standish, & Baum, 1982
Professionally Lead	Family	Adult	Y	UC	334/161*	<1–180	7–70 days; up to 10 sessions	Medical Illness (CMI, MMPI); Psychiatric Illness (Boston Bereavement, Mood Inventory); Family Functioning (Ferriera-Winter, Bodin Drawing); Crisis Coping (Intrapersonal, Family, Job/Financial, Social); Social Cost (Gross Income, Living Expenses, Absenteeism, Economic Loss)	Williams, Lee, & Polak, 1976 Polak, Egan, et al, 1975
Professionally Lead	Family	Adult	Y	UC	176/86*	<1–180	7–70 days; up to 10 sessions	Neurotic Symptoms Scale; Bodin Family Closeness; Crisis Coping Scale; Religious helping of others; Authoritarian Family Functioning; Depression; Monthly Income; Monthly Expenses; Social Costs; Bereavement Adjustment	Williams & Polak, 1979
Professionally Lead	Family	Adult	N	NA	77/37*	<1	>360 days	Personal and social phenomena of death (structured interview)	Oliver, Sturtevant, et al, 2001
Professionally Lead	Family	Child	Y	Y-NE	72/55	<730	15 sessions	Depression (CDI, CBCL, PERI Demoralization Scale); Parental Warmth (CRPBI); Family Cohesion (Family Environment Scale); Parent perception of support (author-created scale); Family Coping (F-COPES)	Sandler, West, et al, 1992
Professionally Lead	Group	Senior	N	NR	28/11	90–7300	<140 days; up to 20 sessions	Social Support (ASSIS); Affect/Mood (PANAS); Emotional/Social Loneliness (ESLI)	Stewart, Craig, et al, 2001
Professionally Lead	Group	Adult	Y	Y	197/166	<180	70 days; 10 sessions	Grief (TRIG, GRI); Distress (POMS); Depression and Anxiety (SIGH-AD)	Goodkin, Blaney, et al, 1999
Professionally Lead	Group	Adult	Y	Y-NE	242/185	0–35	77 days; 3 sessions	Distress (POMS-TMD, Anxiety-tension, Depression-dejection, Anger-hostility, Confusion-bewilderment, Overall emotional disturbance); Self-Esteem (Rosenberg 1965 scale)	Swanson, 1999
Professionally Lead	Group	Adult	Y	Y-NE	150/120	30–240	270 days	Depression (CES-D, BDI); Anxiety (A-Sta); Somatic Symptoms (SOM); Emotional Symptoms (EMOT); Life Satisfaction (Lsat, SelfAnch)	Kay, Guernsey de Zapien, et al, 1993
Professionally Lead	Group	Adult	Y	Y-NE	119/119	<180	70 days; up to 10 sessions	Immunological measures (CD3+CD4+ cell count, CD3+CD8+ cell count, CD4/CD8 ratio, CD3+ cell count, CD4 cell count, Lymphocyte count, T-lymphocyte count); Neuroendocrine measure (Plasma cortisol level)	Goodkin, Feaster, et al, 1998
Professionally Lead	Group	Adult	Y	Y-NE	110/80	~730	28 days; 8 sessions	Coping and Adaptation (TAT)	Balk, Lampe, et al, 1998
Professionally Lead	Group	Adult	Y	Y-NE	36/36	<180	70 days; up to 10 sessions	Plasma Viral Load (HIV-1 RNA copy number)	Goodkin, Baldewicz, et al, 2001
Professionally Lead	Group	Adult	Y	N	159/127	42–140	Up to 25 sessions	Social Support (SSES); Group Involvement (Liberman & Videka-Sherman, 1986); Depression (CES-D, POMS-D); Anger (POMS-A); Anxiety (POMS-T); Stress (IES)	Levy, Derby, et al, 1993
Professionally Lead	Group	Adult	Y	N	121	30–4745	30–365 days	Grief (HGRC subscales: Despair, Panic behavior, Personal growth, Blame and Anger, Detachment, Disorganization)	DiMarco, Menke, & McNamara, 2001
Professionally Lead	Group	Adult	N	Y	139/107	90–17155	84 days; 12 sessions	Avoidance/Intrusion (IES); Grief (TRIG); Interpersonal Distress (IIP); Social Functioning (SAS-SR); Depression (BDI); Anxiety (STAI); Mental Distress (BSI, GSI); Self-Esteem (SES); Physical Functioning (SF-36); Symptomatic Distress (SCL-90)	Piper, McCallum, et al, 2001**
Professionally Lead	Group	Adult	N	N	83/70	<30–8030	49 days; 7 sessions	Physical, Emotional, and Social Functioning (author created measures); Self Esteem (Rosenberg, 1962); Locus of Control (I-E); Life satisfaction (Neugarten, Havighurdt, & Tobin, 1961); Attitude Toward Women (Spence & Helmreich, 1972, Gump 1972)	Barrett, 1978
Professionally Lead	Group	Adult	N	NA	392/77	NR	56 days	Distress (BSI); Group Process and Satisfaction (author created questionnaire)	Glajchen & Magen, 1995
Professionally Lead	Group	Adult	N	NA	174/138	780	730 days	Motives for joining (Lieberman 1979); Interpersonal relations (Porat 1987); Group leadership style (Porat 1987); Perceived contribution of treatment on recovery	Geron, Ginsberg, & Solomon, 2003
Professionally Lead	Group	Adult	N	NA	21/21	NR	70 days; up to 10 sessions	Perceived Social Support (PRQ); Perceived Stress (PSS)	Davis, Hoshiko, et al, 1992
Professionally Lead	Group	Adult	N	NA	21/21	365–3650	52 days; up to 8 sessions	Depression (CDI); Anxiety (HSC-25); Knowledge of Death and Bereavement (KDBQ)	Stoddart, Burke, & Temple, 2002
Professionally Lead	Group	Adult	N	NA	20	90–1095	<1095 days; unlimited sessions	Grief (TRIG); Social Network (SNM, SNG)	Forte, Barrett, & Campbell, 1996
Professionally Lead	Group	Adult	N	NA	12/5	60–780	28 days; 8 sessions	Emotional Distress (EPI); Family Adjustment (FACES-III); Social Adjustment (SAS-SR)	Heiney, Ruffin, & Goon-Johnson, 1995
Professionally Lead	Group	Child	Y	Y-NE	17/17	>730	42 days; 6 sessions	Self-Esteem (PH); Depression (CDI); Behavior (CBCL-TRF, CBCL-YSR)	Huss & Ritchie, 1999
Professionally Lead	Group	Child	N	NA	38/29	<900	300 days; 12 sessions	Depression (BID); Attitude/ Conception of Death (ATCD)	Shilling, Koh, et al, 1992
Professionally Lead	Group	Child	N	NA	18/18	<730	52 days; 8 sessions	Bereavement Survey (author created); Loss Resolution (LRS-Modified); Distress and Somatic Complaints (ALAC)	Opie, Goodwin, Finke, et al, 1992
Professionally Lead	Group	Child	N	NA	6/6	240–1020	42 days; 6 sessions	Psychological measures (Lewis Counselling Inventory, IPAT)	Quarmby, 1993
Professionally Lead	Group	Child	N	NA	4/4	<90	77 days; 11 sessions	Self-Esteem (Piers-Harris Self-Concept Scale); Descriptive (Risk Impact, Negative Chain Events, Opening Up Opportunities)	Zambelli & DeRosa, 1992
Professionally Lead	Couple/ Marital	Adult	Y	Y-NE	57/31	NR	Mean of 6 sessions	Grief (TRIG); Irritability, Depression, Anger (IDA)	Lilford, Stratton, et al, 1994

Notes: * Families, not individuals. ** Study also included psychotherapy condition. Legend: Type, Type of Intervention; Format, Format of Intervention; Pop, Target Population; CG, Control Group; RA, Random Assignment; Num, Number of subjects; TSL, Time Since Loss; DT, Duration of Trial; NA, Not Applicable; NR, Not Reported; UC, Unclear; Y, Yes; N, No; Y-NE, Randomization mentioned, but allocation method not explicitly stated; RS, Randomization Subverted.

Several studies documented substantial spontaneous improvements in bereavement symptomology in the control groups. Kay and others (1993) report a bereavement intervention for Mexican-American widows [33]. They found that all widows improved on all depression scales, state anxiety, life satisfaction, and emotional and somatic symptom scales over the course of two years. However, those widows in the experimental support group exhibit significantly improved changes in these scores. Tudiver and colleagues (1992) conducted a mutual-help support group for recently bereaved widowers [17] that can be compared to Vachon and colleagues' (1980) and Barrett's (1978) widow studies [14,39]. Tudiver and others found significant improvement over time (baseline to eight months) for all widowers, but found no significant differences between those who received treatment and a comparison group of windowers who were on the wait list to receive treatment but had not.

***Psychotherapy-based treatments***, another form of psychological interventions, can be done in different formats (family, group, or individual), and via different approaches. Of the 25 studies that use psychotherapy as an intervention, approaches included cognitive-behavioral, psychodynamic, psychoanalytical, and interpersonal approaches, as well as combinations of these and modality and social support (Table 3)[6,19,22,35,38,53-72]. Seventeen of these studies utilized control groups, only 13 claimed randomization, and only five of these clearly stated their method of allocation.

**Table 3:** Psychotherapy Interventions

**Type**	**Format**	**Pop**	**CG**	**RA**	**Num**	**TSL (days)**	**DT**	**Key Outcome Measures**	**Article**
**Cognitive-behavioral**	Individual	Senior	Y	N	58/NR	120–180	70 days; 4 sessions	Mastery (Personal Mastery Scale); Well-being (MHI subscales, ABS Subscale, PERI self-esteem); Distress (PERI Demoralization Scales, MHI subscales)	Reich & Zautra, 1989
	Individual	Senior	N	NA	4/4	540–730	98 days; 14–18 sessions	Distress (SUDS); Grief (ICG); Depression (BDI); Anxiety (BAI)	Harkness, Shear, et al, 2002**
	Individual	Adult	Y	Y	30/25	>90	35 days; 10 sessions	Avoidance/Intrusion (IES); Anxiety (SCL-90); Depression (SCL-90); Mood (POMS)	Lange, van de Ven, et al, 2001
	Individual	Adult	Y	Y-NE	26/14	180–7300	70 days; 6 sessions	Depression (Wakefield, BDI); Physical Symptoms (Mawson et al, 1981); Fear (FQ); Grief (TRIG); Avoidance (Bereavement Avoidance Tasks)	Sireling, Cohen, & Marks, 1988
	Group	Adult	Y	Y-NE	261/147	46–229	84 days; 8 sessions	Mental Distress (BSI, GSI); PTS Symptoms (TES); Grief (GES); Physical Health (HHB); Marital Strain (DAS)	Murphy, Johnson, et al, 1998 Murphy, 1997
	Group	Adult	Y	Y-NE	110/80	~730	28 days; 8 sessions	Coping and Adaptation (TAT)	Balk, Lampe, et al, 1998
	Group	Adult	Y	N	38/21	90–750	70 days; 10 sessions	Treatment Expectancy (Expectancy Scale); Depression (BDI); Avoidance, Anxiety (Social Anxiety and Distress Scale); Enjoyability (Pleasant Events Scale); Life Satisfaction (Life Satisfaction Scale)	Walls & Meyers, 1985
	Group	Adult	N	NA	8/8	>30	56 days; 8 sessions	Avoidance/Intrusion (IES); Depression (BDI, SCL-90-R); Anxiety (SCL-90-R, STAI); Grief (GRI); Distress (PERI Demoralization)	Sikkema, Kalichman, et al, 1995****
	Group	Child	Y	UC	19/18	<730	NR	Behavior (BRIC-S, BRIC-H); Depression (DSRS); Grief (BP)	Hilliard, 2001
**Psycho-dynamic**	Individual	Senior	Y	Y	228	~60	<180 days; Unlimited sessions	Number of Office Visits, Types of Illnesses	Gerber, Wiener, Battin, et al, 1975
	Individual	Senior	Y	Y-NE	33/30	90–1170	14 days; 4 sessions	Mental Distress (BSI); Depression (GDS); Hopelessness (GHS); Avoidance/Intrusion (IES); Mood (PANAS)	Segal, Bogaards, et al, 1999
	Individual	Adult	Y	Y	66/56	<49	90 days; up to 9 sessions	General Health(general health questionnaire)	Raphael, 1977
	Individual	Adult	Y	N	72/63	60–462	12–20 sessions	Avoidance/Intrusion (IES-A, IES-I); Depression (SCL-90); Anxiety (SCL-90); Total Pathology (SCL-90); Stress-Intrusion (SRRS); Neurotic Symptoms (BPRS)	Horowitz, Weiss, et al, 1984
	Individual	Adult	N	Y-NE	12/6	365–3650	196 days	Depression (Wakefield); Grief (TRIG) Phobic Avoidance (FQ); Hostility/Anger/Guilt (HAG); Attitude to self and deceased (author-created scales); Avoidance (Bereavement Avoidance Tasks); Physical Symptoms (Maddison & Viola, 1968); Compulsive Behavior (Compulsive Activity Checklist); Social Adjustment (Watson & Marks, 1971)	Mawson, Marks, et al, 1981
	Individual	Adult	N	NA	1/1	<180	112 days; 10 sessions	Grief (Grief Scale); Coping (CRI)	Orton, 1994
	Group	Senior	Y	Y	150/117	<365–7300	540 days; 6 sessions	Depression (BDI); Socialization (RSAS)	Constantino, 1988*****
**Psycho-dynamic**	Group	Adult	Y	Y-NE	56/53	120–330	8 sessions	Depression, Anxiety, Somatization (Hopkins Symptom Checklist); Grief Intensity, Preoccupation, Guilt, Anger (Lieberman & Videka-Sherman, 1986); Psychological Distress (Bradburn Affect Balance Scale); Locus of Control (Pearlman et al, 1981); Self-Esteem (Rosenberg scale, 1965); Social Adjustment (Pearlman et al, 1981, Lieberman & Videka-Sherman, 1986)	Lieberman & Yalom, 1992
	Group	Adult	Y	N	50/50	NR	90 days; 14 sessions	Grief (TRIG)	Sabatini, 1988–89
	Group	Adult	N	Y	139/107	90–17155	84 days; 12 sessions	Avoidance/Intrusion (IES); Grief (TRIG); Interpersonal Distress (IIP); Social Functioning (SAS-SR); Depression (BDI); Anxiety (STAI); Mental Distress (BSI, GSI); Self-Esteem (SES); Physical Functioning (SF-36); Symptomatic Distress (SCL-90)	Piper, McCallum, et al, 2001*
	Group	Adult	N	Y-NE	61/55	120–1095	84 days; 12 sessions	Avoidance/Intrusion (IES); Stress Symptoms (SRRS); Depression (BDI); Mental Distress (BPRS, SCL-90); Social Functioning (SAS-SR); Overall Functioning (GAS)	Marmar, Horowitz, et al, 1988**
	Group	Child	Y	N	16/16	30–365	56 days	Depression (CDI); Behavior (AP, AT); Grief (BP); Family alliance (TP); Grief, family relationship (TC)	Tonkins & Lambert, 1996
	Group	Child	N	NA	45/37	30–3650	70 days	Trauma (CPTSRI)	Salloum, Avery, & McClain, 2001
**Psycho-analytic**	Group	Adult	N	N	154/59	NR	84 days; 12 sessions	Affect (author created); Psychodynamic Work (PWORS); Severity of objectives (author created)	McCallum, Piper, & Morin, 1993
**Inter-personal**	Individual	Senior	Y	Y-NE	80/66	216–279	112 days; up to 16 sessions	Grief (TRIG)	Reynolds, Miller, et al, 1999***
**Behavioral and Psycho-dynamic**	Individual	Adult	Y	N	83/83	<1825	15–20 sessions	Anger (State Trait Anger Inventory); Anxiety (STAI); Avoidance/Intrusion (IES); Somatic/psychoneurotic symptoms (SCL-90); (Locus of Control Scale)	Kleber & Brom, 1987

Notes: * Study also included support/counselling condition. ** Treatment also included aspects of interpersonal psychotherapy. *** Study also included pharmacotherapy condition. **** Treatment also included aspects of social support. ***** Study also included social activities condition. Legend: Type, Type of Intervention; Format, Format of Intervention; Pop, Target Population; CG, Control Group; RA, Random Assignment; Num, Number of subjects; TSL, Time Since Loss; DT, Duration of Trial; NA, Not Applicable; NR, Not Reported; UC, Unclear; Y, Yes; N, No; Y-NE Randomization mentioned, but allocation method not explicitly stated.

***Cognitive-behavioral therapy ***was employed in nine trials, four of which used individual sessions while five studies used group sessions. Murphy and colleagues (1998) studied an intervention for parents bereaved by the violent death of their children [57]. The results show no treatment effect between intervention and control groups over the five main tested outcome variables. The authors then proceeded with a post-hoc subgroup analysis, which identified mothers with high Global Severity Index scores and grief at baseline as potentially benefiting from intervention during the period, while fathers who received the intervention appeared to have more posttraumatic stress disorder (PTSD) symptoms at six-month follow-up.

Kleber and Brom (1987) conducted a comparative outcome study of three forms of short-term psychotherapy [69]. They compared the results of 83 patients suffering from a major loss who had been randomized into hypnotherapy (behavioral), trauma desensitization (behavioral), psychodynamic therapy, and a delayed-treatment control group. They found all three therapies successful in improving patients' conditions, but did not find any particular therapy to be significantly more effective than another. While the control group showed slight recovery, over time the three therapies were more effective in reducing symptoms of the bereavement response.

Studies of ***psychodynamic ***therapy, which strives for the patient to understand and cope better with feelings by re-experiencing them and talking them through with the aid of the therapist, was found to be quite prevalent in the bereavement care literature. Overall, the results are mixed, with more support found in the group format of psychodynamic therapy than in individual therapy. Of the studies we evaluated as psychodynamic therapy, six were individual in format, seven had a group format, and eight employed control groups; five of these claimed random allocation (one additional study randomly assigned subjects to two experimental conditions but lacked a control group).

***Psychoanalysis***, as exemplified by Freud, proceeds with an inward investigation of unconscious mental processes and childhood experiences as the principal therapeutic procedure. Problematic measurement methodology beset the one study that utilized a group format to provide a psychoanalytic-based intervention (with no details regarding the tasks assigned to the patients)[68]. This study focused primarily on the relationship between the patient's personal affect (measured by an unvalidated affect assessment scale) and a favorable treatment outcome (measured again by an ad-hoc unvalidated measure).

***Behavioral therapy ***uses learning principles (such as behavior modification, systematic desensitization, and aversion) to eliminate or reduce unwanted reactions to either external situations, one's thoughts and feelings, and bodily sensations or functions. Behavioral therapy was used in only one study, which compared traumatic desensitization to hypnotherapy and psychodynamic therapies [69]. As described in the section on cognitive-behavioral therapies above, all three therapies resulted in significant improvements from pre- to post-treatment as compared to controls, and no one therapy was found to be more effective than the others in treating bereavement-related symptoms.

***Interpersonal therapy ***aims to improve communication skills and increase self-esteem during a short time period by focusing on a patient's behaviors and social interactions with family and friends, directly teaching how better to relate to others. Only one study used interpersonal therapy as a bereavement care intervention, and this study found no effect on grief as the only measured outcome [6].

### Systems-oriented interventions

Seven studies featured interventions that altered the manner in which the healthcare system interacted with patients, family, and friends prior to death, guided by an underlying (yet not fully explicated) notion that interactions experienced by loved ones prior to death can influence the subsequent bereavement process (Table 4) [73-79]. Six of the seven interventions provided enhanced or augmented care, in the form of palliative care, hospice care, or care coordination. One intervention gave family members the option of witnessing resuscitation efforts [79]. Overall, the studies that reported systems-oriented interventions produced mixed results of efficacy, with only three of the seven studies showing any treatment effect, mostly in long-term follow-up ranging from 60–365 days post-death. In fact, no study found significant treatment effects when measured during the intervention.

**Table 4:** Systems-Oriented Interventions

**Intervention**	**Pop**	**CG**	**RA**	**Num**	**Time of Evaluation**	**Key Outcome Measures**	**Article**
Care Coordination	Relative of cancer death	Y	Y	94	365 days pre-death 56 days post-death	Anxiety (HADA, Leeds Depression and Anxiety Scale); Depression (HADD, Leeds Depression and Anxiety Scale); Social Support (Family Apgar Scale)	Addington, MacDonald, et al, 1992
Emergency Room	Relative of Emergency Room Death	Y	N	100/66	180–365 days post-death	Changes in satisfaction of care, information received (author-created questionnaire)	Adamowski, Dickinson, et al, 1993
Hospice Care	Relative of cancer death	Y	Y-NE	96	42 days post-death 540 days post-death	Depression (CES-D); Anxiety (Rand Health Insurance Study); General Health (Rand Health Insurance Study); Social Functioning	Kane, Klein, et al, 1986
Palliative Care	Relative of cancer death	Y	Y	183	60–270 days pre-death 390 days post-death	Grief (TRIG2)	Ringdal, Jordhoy, et al, 2001
	Relative of cancer death	Y	NR	119/49	0–60 days post-death	Anxiety, Depression, Mental Exhaustion ("observations and ratings")	Haggmark & Theorell, 1988
	Relative of cancer death	Y	N	49/37	365 days post-death	Health, Anger, Mental State, Depression (Holland & Segroi's instrument)	Haggmark, Bachner, & Theorell, 1991
Witnessed Resuscitation	Relative of unsuccessful resuscitation	Y	Y	18	30 days post-death 90 days post-death	Grief (TRIG1, TRIG2); Avoidance/Intrusion (IESA, IESI); Depression (BDI, HADD); Anxiety (HADA, BAI)	Robinson, Makenzie-Ross, et al, 1998

Legend: Pop, Target Population; CG, Control Group; RA, Random Assignment; Num, Number of subjects; NR, Not Reported; Y, Yes; N, No; Y-NE Randomization mentioned, but allocation method not explicitly stated.

Ringdal and colleagues (2001) found no significant differences between those family members whose relative received palliative care and those who received traditional care [76]. This intervention, however, was not directed to the bereaved relatives, but rather to their terminally ill relatives. The bereaved relatives did show an overall significant decline in TRIG grief scores over one year post-bereavement for both palliative and traditional care groups.

Robinson (1998) examined the psychological effect of witnessing resuscitation efforts of patients in the emergency room on bereaved relatives [79]. They found no psychological differences between the control group who did not witness the resuscitation attempt and the experimental group who had the option of viewing the resuscitation effort. In fact, at the three- and nine-month follow up, the experimental group exhibited median scores lower (that is, better) than the controls on five of the eight measured scales. At nine months, the authors found the difference in TRIG2 scores approaching the 5% significance level with a reported p = 0.08. These findings provide no evidence to support the popular belief that relatives should be excluded from the resuscitation room, and provide only weak evidence of possible psychological benefit of witnessed resuscitation; they do not, however, suggest that having witnessed an unsuccessful resuscitation attempt alleviates the grief reaction of the bereaved.

## Discussion

When reviewed systematically, the current bereavement intervention literature – notwithstanding the existence of many intriguing reports – yields few reliable conclusions to guide treatment. Good evidence supports the pharmacological treatment of depression occurring in the context of bereavement. For all other forms of intervention, however, and for all attempts to diminish grief *per se*, no consistent pattern of treatment benefit has been established across well-designed experimental studies.

Why – despite prevalence of bereavement, the intense dedication on the part of the bereavement research community, and the multitude of peer-reviewed published bereavement studies – does the field of bereavement care lack a formidable evidence base? In order to improve the effectiveness and quality of bereavement care, this question begs to be addressed. On the basis of our systematic review of the literature, we postulate the following five factors as hindering methodical scientific progress regarding bereavement care interventions.

### Excessive theoretical heterogeneity

As the history of science and medicine suggests, successful scientific inquiry into a topic is typically a cumulative process undertaken by a community of investigators working within a shared scientific paradigm [80,81]. The field of bereavement care intervention studies does not appear to be organized in such a manner, but instead consists of distinct groups of investigators working within disparate theoretical frameworks: pharmacologic, psychodynamic, psychoanalytic, behavioral, cognitive-behavioral, interpersonal, and social supportive theories each vie for attention. Indeed, although specification of an underlying treatment-theory conceptual model may improve causal inference [82], the bereavement care literature may be too invested in and reliant on theoretical justifications of treatments. Consequently, the compiled published reports demonstrate a cumulative 'Tower of Babel' phenomenon, with the different theory-dominated perspectives failing to engage each other meaningfully: the sum is no greater than the parts, and perhaps less.

### Stultifying between-study variation

Treatments featured in published studies vary almost as much as the authors who tested them. One can observe substantial variation across studies regarding the type of intervention generally or regarding the specific implementation of a specific type of intervention (such as different doses of pharmaceuticals); regarding characteristics of targeted patient populations; regarding outcome measurements and study design methodology. Scrutinizing the key outcome measures listed in the accompanying tables illustrates this remarkable heterogeneity. Although these differences have been due in part to diverse treatment-theory paradigms, even studies conducted within the same theoretical paradigm often differed markedly in terms of what potential benefit was being tested, and how it was being measured. Such substantial variation between studies stymies comparison or confirmation of treatment effects.

### Inadequate reporting of intervention procedures and implementation

Aside from the pharmacological studies, which reported the dosing of the intervention medication, very few reported studies describe the intervention procedures in sufficient detail for readers to envision clearly what tasks or activities intervention subjects were asked to perform. This under-specification prevents sensible analysis, within a class of treatments (such as cognitive-behavioral therapy), of observed differences in treatment effects (since the implementation of cognitive-behavioral therapy, for instance, may have been quite different in seemingly similar intervention studies).

### Few published "replication" studies

Inadequate specification of intervention procedures, combined with other factors at work within the community of bereavement care investigators, may have resulted in the dearth of published replication studies. This lack of replication prevents the accumulation of a body of evidence that would confirm, refute, or refine prior estimates of treatment effects.

### Methodological study-design and data-analysis flaws

A final factor inhibiting research progress in the realm of bereavement care interventions encompasses a number of recurring methodological flaws that greatly limit inferences regarding treatment effects. First and foremost is the omission of control groups. Control groups are essential for the valid evaluation of a bereavement intervention, particularly because of the typically self-limited course of grief: even absent any treatment, most bereaved people show "diminished pathological symptoms and fewer signs of disturbance within two years of the loss"[65]. Purported beneficial treatment effects observed in an intervention group without a suitable control group therefore may in fact be simply the natural grief remission process. A second common study design feature is the non-random assignment of study subjects into treatment and control groups, which again limits the strength of inference regarding observed 'treatment' effects, as these differences between treatment and control groups may be due to selection or assignment bias. Third, many studies measured subject outcomes using untried assessment tools that had been created on an *ad hoc *basis, and which may therefore have compromised measurement accuracy and inference validity. Lastly, studies that failed to demonstrate a statistically-significant difference for the main outcome measure often performed numerous *post hoc *subgroup analyses, a practice that negates the rigor of statistical hypothesis testing.

If these five factors are indeed hampering progress towards improving bereavement care interventions and quality of care for bereaved individuals, then concrete actions could facilitate progress within the field of bereavement care, specifically: 1) Convening a consensus-building conference among key stakeholders and investigators to define a specific research agenda that would draw on a limited number of theoretical paradigms and delineate key elements of treatment theory [82]; 2) Focusing on interventions to improve key outcomes that are valued by bereaved individuals; 3) Targeting well-defined patient populations at well-defined phases of bereavement; 4) Conducting high-quality randomized controlled trial research designs, employing rigorous tests of hypotheses defined prior to the conduct of the study, and eschewing unplanned subgroup analyses; 5) Weighing the ethical arguments for and against the use of randomized control subjects in such research; 6) Increasing incentive to conduct and publish highly-comparable replication studies; and 7) Enforcing the adoption of uniform standards regarding clinical trial study reporting (such as outlined in the CONSORT statement [83]) by journal editors and the bereavement research community.

## Competing interests

None declared.

## Abbreviations

CG Control group

NT Nortriptyline

SSRI Selective serotonin reuptake inhibitors

TCA Tricyclic antidepressants

TRIG Texas Revised Inventory of Grief

## Authors' contributions

AF assisted in the design of the review, conducted data collection, data abstraction, and drafted the manuscript. MH assisted in the design of the review and critically reviewed several drafts of the manuscript. RP assisted in data collection and abstraction. CF conceived of and designed the review, assisted in drafting and revision of the manuscript, and provided support and mentorship through the process. All authors read and approved the final manuscript.

## Pre-publication history

The pre-publication history for this paper can be accessed here:



## Supplementary Material

Additional File 1 All citations. This file contains citations to all of the studies identified by our literature search and screened for inclusion in this review, as well as other scholarly works consulted during the conduct of this review. Click here for file
