# Supplementary material for: Bereavement care interventions: a systematic review
Source: BMC Palliat Care. 2004 Jul 26;3:3. doi: 10.1186/1472-684X-3-3 (PMC503393; doi:10.1186/1472-684X-3-3)
Supplement: Additional File 1 — All citations. This file contains citations to all of the studies identified by our literature search and screened for inclusion in this review, as well as other scholarly works consulted during the conduct of this review. [file 1472-684X-3-3-S1.pdf]

BEREAVEMENT INTERVENTION SYSTEMATIC REVIEW: LIST OF REFERENCES FOUND, AND ADDITIONAL REFERENCES USED

1970 (1)

1. Kuhn T: **The structure of scientific revolutions**, 2nd edn. Chicago: University of Chicago Press; 1970.

1975 (2)

1. Gerber I, Wiener A, Battin D, Arkin A: **Brief therapy for the bereaved**. In: *Bereavement: Its psychosocial aspects.* Edited by Shoenberg B, Gerber I. New York: Columbia University Press; 1975: 310-333.
2. Polak PR, Egan D, Vandenbergh R, Williams WV: **Prevention in mental health: a controlled study**. *American Journal of Psychiatry* 1975, **132**(2):146-149.

1976 (1)

1. Williams WV, Lee J, Polak PR: **Crisis intervention: effects of crisis intervention on family survivors of sudden death situations**. *Community Mental Health Journal* 1976, **12**(2):128-136.

1977 (2)

1. Anonymous: **Newcastle survey of deaths in early childhood 1974/76, with special reference to sudden unexpected deaths. Working party for early childhood deaths in Newcastle**. *Archives of Disease in Childhood* 1977, **52**(11):828-835.
2. Raphael B: **Preventive intervention with the recently bereaved**. *Archives of General Psychiatry* 1977, **34**(12):1450-1454.

1978 (1)

1. Barrett AV: **Effectiveness of widows' groups in facilitating change**. *Journal of Consulting and Clinical Psychology* 1978, **46**(1):20-31.

1979 (2)

1. Fleck L: **Genesis and development of a scientific fact**. Chicago: University Press; 1979.
2. Williams WV, Polak PR: **Follow-up research in primary prevention: a model of adjustment in acute grief**. *Journal of Clinical Psychology* 1979, **35**(1):35-45.

1980 (3)

1. Miles MS: **The effects of a course on death and grief on nurses' attitudes toward dying patients and death**. *Death Education* 1980, **4**(3):245-260.
2. Parkes CM: **Bereavement counselling: does it work?** *Br Med J* 1980, **281**(6232):3-6.
3. Vachon ML, Lyall WA, Rogers J, Freedman-Letofsky K, Freeman SJ: **A controlled study of self-help intervention for widows**. *American Journal of Psychiatry* 1980, **137**(11):1380-1384.

1981 (3)

1. Aries P: **The Hour of Our Death**. Oxford: Oxford University Press; 1981.
2. Dimond M: **Bereavement and the elderly: a critical review with implications for nursing practice and research**. *Journal of Advanced Nursing* 1981, **6**(6):461-470.
3. Mawson D, Marks I, Ramm L, Stern R: **Guided mourning for morbid grief: a controlled study**. *British Journal of Psychiatry* 1981, **138**:185-193.

1982 (2)

1. Forrest GC, Standish E, Baum JD: **Support after perinatal death: a study of support and counselling after perinatal bereavement**. *British Medical Journal Clinical Research Ed* 1982, **285**(6353):1475-1479.
2. Rogers J, Sheldon A, Barwick C, Letofsky K, Lancee W: **Help for families of suicide: survivors support program**. *Canadian Journal of Psychiatry - Revue Canadienne de Psychiatrie* 1982, **27**(6):444-449.

1983 (1)

1. Segantini A, Carnelli V, Portaleone D, Zerbi Schwartz L, Falzi G, Salice P, Schwartz PJ, Careddu P: **Sudden infant death syndrome in Italy. A multidisciplinary approach**. *Pediatria Medica e Chirurgica* 1983, **5**(3):57-60.

1984 (2)

1. Horowitz MJ, Weiss DS, Kaltreider N, Krupnick J, Marmar C, Wilner N, DeWitt K: **Reactions to the death of a parent. Results from patients and field subjects**. *Journal of Nervous & Mental Disease* 1984, **172**(7):383-392.
2. Persson G, Alstrom JE, Nordlund CL: **Prognostic factors with four treatment methods for phobic disorders**. *Acta Psychiatrica Scandinavica* 1984, **69**(4):307-317.

1985 (2)

1. Videka-Sherman L, Lieberman M: **The effects of self-help and psychotherapy intervention on child loss: the limits of recovery**. *American Journal of Orthopsychiatry* 1985, **55**(1):70-82.

2. Walls N, Meyers AW: **Outcome in group treatments for bereavement: experimental results and recommendations for clinical practice**. *International Journal of Mental Health* 1985, **13**(3-4):126-147.

1986 (5)

1. Kane R, Klein S, Bernstein L, Rothenberg R: **The role of hospice in reducing the impact of bereavement**. *Journal of Chronic Diseases* 1986, **39**(9):735-742.
2. Lieberman MA, Videka-Sherman L: **The impact of self-help groups on the mental health of widows and widowers**. *American Journal of Orthopsychiatry* 1986, **56**(3):435-449.
3. Murphy SA: **Stress, Coping, and Mental Health Outcomes Following a Natural Disaster: Bereaved Family Members and Friends Compared**. *Death Studies* 1986.
4. O'Neil MK, Lancee WJ, Freeman SJ: **Psychosocial factors and depressive symptoms**. *Journal of Nervous & Mental Disease* 1986, **174**(1):15-23.
5. Warnes H: **Alexithymia, clinical and therapeutic aspects**. *Psychotherapy & Psychosomatics* 1986, **46**(1-2):96-104.

1987 (7)

1. Burgess C: **Stress and cancer**. *Cancer Surveys* 1987, **6**(3):403-416.
2. Dyregrov A, Matthiesen SB: **Similarities and Differences in Mothers' and Fathers' Grief following the Death of an Infant**. *Scandinavian Journal of Psychology* 1987.
3. Jacobs SC, Nelson JC, Zisook S: **Treating depressions of bereavement with antidepressants: a pilot study**. *Psychiatric Clinics of North America* 1987, **10**(3):501-510.
4. Kleber RJ, Brom D: **Psychotherapy and pathological grief controlled outcome study**. *Israel Journal of Psychiatry & Related Sciences* 1987, **24**(1-2):99-109.
5. Lehman DR, Wortman CB, Williams AF: **Long-term effects of losing a spouse or child in a motor vehicle crash**. *Journal of Personality & Social Psychology* 1987, **52**(1):218-231.
6. Steinberg JA, Silverman MM (eds.): **Preventing mental disorders: A research perspective**; 1987.
7. Wasow M, Coons DH: **Widows and Widowers of Alzheimer's Victims: Their Survival after Spouses' Death**. *Journal of Independent Social Work* 1987, **2**(2):21-32.

1988 (6)

1. Constantino RE: **Comparison of two group interventions for the bereaved**. *Image - the Journal of Nursing Scholarship* 1988, **20**(2):83-87.
2. Gottlieb BH (ed.): **Marshaling social support: Formats, processes, and effects**; 1988.

3. Haggmark C, Theorell T: **Evaluation of an activation programme: repeated observations of mental exhaustion, anxiety and depression in relatives of cancer patients**. *Scand J Caring Sci* 1988, **2**(3):129-141.
4. Kenner CA: **Parent transition from the newborn intensive care unit (NICU) to home**. *D.n.s.*: INDIANA UNIVERSITY SCHOOL OF NURSING; 1988.
5. Marmar C, Horowitz M, Weiss D, Wilner N, Kaltreider N: **A controlled trial of brief psychotherapy and mutual-help group treatment of conjugal bereavement**. *American Journal of Psychiatry* 1988, **145**(2):203-209.
6. Sireling L CD, Marks I: **Guided mourning for morbid grief: A controlled replication**. *Behavior Therapy* 1988, **19**(2):121-132.

1989 (7)

1. Carroll RM: **The relationship of bereaved parental distress, coping, family functioning, cohesiveness and spousal support with infant death**. *Ph.d.*: UNIVERSITY OF MARYLAND AT BALTIMORE; 1989.
2. Fuller RL, Geis SB, Rush J: **Lovers and significant others**. In: *Disenfranchised grief: Recognizing hidden sorrow (pp 33-42).* Edited by Doka KJ, vol. xvi,; 1989: 347.
3. Kim CJ, Yoo JS, Park JW: **The effect of crisis intervention by the visiting nurse with cancer patients**. *Kanho Hakhoe Chi [Journal of Nurses Academic Society]* 1989, **19**(1):63-80.
4. Maton KI: **The Stress-Buffering Role of Spiritual Support: Cross-Sectional and Prospective Investigations**. *Journal for the Scientific Study of Religion* 1989, **28**(3):310-323.
5. Mulligan JCA: **Dying at home: an evaluation of a specialist home care service**. *Ph.d.*: UNIVERSITY OF WALES (UNITED KINGDOM); 1989.
6. Ramsey CN, Jr. (ed.): **Family systems in medicine**; 1989.
7. Reich JW, Zautra AJ: **A perceived control intervention for at-risk older adults**. *Psychology & Aging* 1989, **4**(4):415-424.

1990 (7)

1. Ahmedzai S: **Measuring quality of life in hospice care**. *Oncology (Huntington)* 1990, **4**(5):115-119; discussion 129.
2. Clark EJ: **Sociological Approaches to Illness and Loss**. In.: International Sociological Association; 1990.
3. Gordon SB: **Adolescent sexuality: Treatment of the sleeper effects of sexual abuse**. In: *Adolescent behavior therapy handbook Springer series on behavior therapy and behavioral medicine, (pp 233-252).* Edited by Feindler EL, Kalfus GR, vol. 22; 1990: 459.
4. Koomen W, Kniesmeijer T, Vospanhuijsen A, Velthuijsen AS: **Social Support and Well-Being in Heart-Patients - a Longitudinal-Study of the Combined Role of Need for Social Support and Perceived Social Support**. *Social Behaviour* 1990, **5**(5):297.

5. McCallum M, Piper W: **A controlled study of effectiveness and patient suitability for short-term group psychotherapy**. *International Journal of Group Psychotherapy* 1990, **40**(4):431-452.
6. Schonfeld DJ, Kappelman M: **The impact of school-based education on the young child's understanding of death**. *Journal of Developmental & Behavioral Pediatrics* 1990, **11**(5):247-252.
7. Welch M: **Trauma recovery: an ethnography**. *Ph.d.*: THE UNIVERSITY OF CONNECTICUT; 1990.

1991 (10)

1. Birnbaum A: **Haven Hugs & Bugs. An innovative multiple-family weekend intervention for bereaved children, adolescents and adults**. *American Journal of Hospice & Palliative Care* 1991, **8**(5):23-29.
2. Finlay I, Dallimore D: **Your child is dead**. *BMJ* 1991, **302**(6791):1524-1525.
3. Geyer S: **Life events prior to manifestation of breast cancer: a limited prospective study covering eight years before diagnosis**. *Journal of Psychosomatic Research* 1991, **35**(2-3):355-363.
4. Haggmark C, Bachner M, Theorell T: **A follow-up of psychological state in relatives of cancer patients one year after the patient's death: effects of an activation program**. *Acta Oncol* 1991, **30**(6):677.
5. He M: **A prospective controlled study of psychosomatic and immunologic change in recently bereaved people**. *Chung-Hua Shen Ching Ching Shen Ko Tsa Chih [Chinese Journal of Neurology & Psychiatry]* 1991, **24**(2):90-93, 124.
6. Levenson JL, Bemis C: **The role of psychological factors in cancer onset and progression**. *Psychosomatics* 1991, **32**(2):124-132.
7. Lewis MA, Hatton CL, Salas I, Leake B, Chiofalo N: **Impact of the Children's Epilepsy Program on parents**. *Epilepsia* 1991, **32**(3):365-374.
8. Pasternak RE, Reynolds CF, 3rd, Schlernitzauer M, Hoch CC, Buysse DJ, Houck PR, Perel JM: **Acute open-trial nortriptyline therapy of bereavement-related depression in late life**. *Journal of Clinical Psychiatry* 1991, **52**(7):307-310.
9. Robinson JH: **A descriptive study of widows' grief responses, coping processes and social support within Roy's adaptation framework**. *Ph.d.*: WAYNE STATE UNIVERSITY; 1991.
10. West SG, Sandler I, Pillow DR, Baca L, Gersten JC: **The use of structural equation modeling in generative research: toward the design of a preventive intervention for bereaved children**. *American Journal of Community Psychology* 1991, **19**(4):459-480.

1992 (21)

1. Addington-Hall JM, MacDonald LD, Anderson HR, Chamberlain J, Freeling P, Bland JM, Raftery J: **Randomised controlled trial of effects of coordinating care for terminally ill cancer patients**. *BMJ* 1992, **305**(6865):1317-1322.
2. Anonymous: **Suspect screening**. *Priorities for Health* 1992, **4**(4):31.

3. Bartrop RW, Hancock K, Craig A, Porritt DW: **Psychological Toxicity of Bereavement - 6 Months after the Event**. *Aust Psychol* 1992, **27**(3):192.
4. Davis JM, Hoshiko BR, Jones S, Gosnell D: **The effect of a support group on grieving individuals' levels of perceived support and stress**. *Archives of Psychiatric Nursing* 1992, **6**(1):35-39.
5. Glass TA, Maddox GL: **The quality and quantity of social support: stroke recovery as psycho-social transition**. *Social Science & Medicine* 1992, **34**(11):1249-1261.
6. Hanson C, Strawser D: **Family presence during cardiopulmonary resuscitation: Foote Hospital emergency department's nine-year perspective**. *Journal of Emergency Nursing* 1992, **18**(2):104-106.
7. Lieberman MA, Yalom I: **Brief group psychotherapy for the spousally bereaved: a controlled study**. *International Journal of Group Psychotherapy* 1992, **42**(1):117-132.
8. Murrell SA, Norris FH, Chipley QT: **Functional Versus Structural Social Support, Desirable Events, and Positive Affect in Older Adults**. *Psychol Aging* 1992, **7**(4):562.
9. Nursten J: **Process following Disaster Work**. *Journal of Social Work Practice* 1992, **6**(2):151-158.
10. Opie ND, Goodwin T, Finke LM, Beattey JM, Lee B, van Epps J: **The effect of a bereavement group experience on bereaved children's and adolescents' affective and somatic distress**. *Journal of Child & Adolescent Psychiatric & Mental Health Nursing* 1992, **5**(1):20-26.
11. Piper WE, McCallum M, Azim HFA: **Adaptation to loss through short-term group psychotherapy**; 1992.
12. Provencio-Vasquez E: **Creating paths: living with a very low birth weight infant**. *Ph.d.*: THE UNIVERSITY OF ARIZONA; 1992.
13. Reynolds CF, 3rd: **Treatment of depression in special populations**. *Journal of Clinical Psychiatry* 1992, **53**(Suppl):45-53.
14. Sandler IN, West SG, Baca L, Pillow DR, Gersten JC, Rogosch F, Virdin L, Beals J, Reynolds KD, Kallgren C *et al*: **Linking empirically based theory and evaluation: the Family Bereavement Program**. *American Journal of Community Psychology* 1992, **20**(4):491-521.
15. Schilling RF, Koh N, Abramovitz R, Gilbert L: **Bereavement Groups for Inner-City Children**. *Research on Social Work Practice* 1992, **2**(3):405.
16. Sherr L, Hedge B, Steinhart K, Davey T, Petrack J: **Unique patterns of bereavement in HIV: implications for counselling**. *Genitourinary Medicine* 1992, **68**(6):378-381.
17. Snow RW, Armstrong JRM, Forster D, Winstanley MT, Marsh VM, Newton C, Waruiru C, Mwangi I, Winstanley PA, Marsh K: **Childhood Deaths in Africa - Uses and Limitations of Verbal Autopsies**. *Lancet* 1992, **340**(8815):351.
18. Solomon SD, Gerrity ET, Muff AM: **Efficacy of Treatments for Posttraumatic-Stress-Disorder - an Empirical Review**. *JAMA-J Am Med Assoc* 1992, **268**(5):633.
19. Teel CSH: **Grief, recurrent sorrow, and depression among caregivers and bereaved**. *Ph.d.*: THE UNIVERSITY OF ARIZONA; 1992.

20. Tudiver F, Hilditch J, Permaul J, McKendree D: **Does mutual help facilitate newly bereaved widowers? Report of a randomized controlled trial**. *Evaluation & the Health Professions* 1992, **15**(2):147-162.
21. Zambelli GC, DeRosa AP: **Bereavement support groups for school-age children: theory, intervention, and case example**. *American Journal of Orthopsychiatry* 1992, **62**(4):484-493.

1993 (15)

1. Adamowski K, Dickinson G, Weitzman B, Roessler C, Carter-Snell C: **Sudden unexpected death in the emergency department: caring for the survivors**. *CMAJ Canadian Medical Association Journal* 1993, **149**(10):1445-1451.
2. Appleton R, Gibson B, Hey E: **The loss of a baby at birth: the role of the bereavement officer.[comment]**. *British Journal of Obstetrics & Gynaecology* 1993, **100**(1):51-54.
3. Caserta MS, Lund DA: **Intrapersonal Resources and the Effectiveness of Self-Help Groups for Bereaved Older Adults**. *Gerontologist* 1993, **33**(5):619.
4. Edmands MS: **Critique of Bereavement reactions among homosexual men experiencing multiple losses in the AIDS epidemic [original article by Neugebauer R et al appears in AM J PSYCHIATR 1992;149(10):1374-9]**. *Nursing Scan in Research* 1993, **6**(5):20-21.
5. Kay M, Guernsey de Zapien J, Wilson C, Yoder M: **Evaluating treatment efficacy by triangulation**. *Social Science & Medicine* 1993, **36**(12):1545-1554.
6. Levy LH, Derby JF, Martinkowski KS: **Effects of membership in bereavement support groups on adaptation to conjuga bereavement**. *American Journal of Community Psychology* 1993, **21**(3):361-381.
7. Lipsey M: **Theory as method: small theories of treatment**. *New Directions for Program Evaluation* 1993, **57**:5-38.
8. McCallum M, Piper WE, Morin H: **Affect and outcome in short-term group therapy for loss**. *International Journal of Group Psychotherapy* 1993, **43**(3):303-319.
9. Pelletier ML: **The needs of family members of organ and tissue donors**. *Heart & Lung: Journal of Acute & Critical Care* 1993, **22**(2):151-157.
10. Pickett M: **Cultural awareness in the context of terminal illness**. *Cancer Nursing* 1993, **16**(2):102-106.
11. Quarmby D: **Peer group counselling with bereaved adolescents**. *British Journal of Guidance and Counseling* 1993, **21**(2):196-211.
12. Rajaram SS, Garrity TF, Stallones L, Marx MB: **Bereavement Loss of a Pet and Loss of a Human**. *Anthrozoos* 1993, **6**(1):8.
13. Robbins RA, McLaughlin NR, Nye-Dameshek J: **Knowledge and beliefs about donation -- I: Asking the experts**. *Journal of Transplant Coordination* 1993, **3**(1):7-13.
14. Short A: **GIM dring pregnancy: anticipation and resolution**. *Journal of the Association for Music & Therapy* 1993, **2**:273-287.

15. Surtees PG, Miller PM: **Partners in adversity: I. Study design and context**. *European Archives of Psychiatry & Clinical Neuroscience* 1993, **242**(4):224-232.

1994 (13)

1. Bledsoe CM: **Factors influencing the decision of families to donate organs**. *Ph.d.*: THE UNIVERSITY OF UTAH; 1994.
2. Crown S, Freeman HL (eds.): **The book of psychiatric books**; 1994.
3. Fabrega H, Jr., Nutini H: **Sudden infant and child death as a cultural phenomenon: a Tlaxcalan case study**. *Psychiatry* 1994, **57**(3):225-243.
4. Feld S, George LK: **Moderating effects of prior social resources on the hospitalizations of elders who become widowed**. *Journal of Aging & Health* 1994, **6**(3):275-295.
5. Hanson JL, Ashley B: **Advanced practice nurses' application of the Stetler model for research utilization: improving bereavement care**. *Oncology Nursing Forum* 1994, **21**(4):720-724.
6. Hays JC, Kasl S, Jacobs S: **Past personal history of dysphoria, social support, and psychological distress following conjugal bereavement**. *Journal of the American Geriatrics Society* 1994, **42**(7):712-718.
7. Kanhai Humphrey HH, Geerinck-Vercammen C, De Haan M, Van Der Ploeg Henk M, Van Zanten Loes A, Bennebroek-Gravenhorst J: **Follow-up of pregnancies, infants, and families after multifetal pregnancy reduction**. *Fertility & Sterility* 1994, **62**(5):955-959.
8. Lilford RJ, Stratton P, Godsil S, Prasad A: **A randomised trial of routine versus selective counselling in perinatal bereavement from congenital disease**. *British Journal of Obstetrics & Gynaecology* 1994, **101**(4):291-296.
9. Mancoske RJ, Standifer D, Cauley C: **The Effectiveness of Brief Counseling-Services for Battered Women**. *Research on Social Work Practice* 1994, **4**(1):53.
10. Orton M: **A Case-Study of an Adolescent Mother Grieving the Death of Her Child Due to Sudden-Infant-Death-Syndrome**. *Am J Art Ther* 1994, **33**(2):37.
11. Pasternak RE, Reynolds CF, Houck PR, Schlernitzauer M, Buysse DJ, Hoch CC, Kupfer DJ: **Sleep in Bereavement-Related Depression During and after Pharmacotherapy with Nortriptyline**. *J Geriatr Psychiatry Neurol* 1994, **7**(2):69.
12. Schneiderman G, Winders P, Tallett S, Feldman W: **Do child and/or parent bereavement programs work?** *Canadian Journal of Psychiatry - Revue Canadienne de Psychiatrie* 1994, **39**(4):215-218.
13. Steptoe A, Wardle J (eds.): **Psychosocial processes and health: A reader**; 1994.

1995 (20)

1. Akers MC: **Transition readiness, grief work, psychological symptom distress and maternal adaptation as components of a life transition**. *D.n.sc.*: THE CATHOLIC UNIVERSITY OF AMERICA; 1995.

2. Balk DE: **Bereavement Research Using Control Groups: Ethical Obligations and Questions**. *Death Studies* 1995, **19**(2):123-138.
3. Bean NM: **Stranger in our home: Rural families talk about the experience of having received in-home family services**. *Dissertation Abstracts International* 1995, **55**(9-A):2986.
4. Blackwelder NL: **Critical incident stress debriefing for school employees**. *Dissertation Abstracts International* 1995, **56**(4-A):1192.
5. Clark SE, Goldney RD: **Grief reactions and recovery in a support group for people bereaved by suicide**. *Crisis: Journal of Crisis Intervention & Suicide* 1995, **16**(1):27-33.
6. Czupek DA: **Physician and family factors which influence the consent process in pediatric organ donation**. *Dissertation Abstracts International: Section B: the Sciences & Engineering* 1995, **55**(8-B):3563.
7. Dieckmann RA, Vardis R: **High-Dose Epinephrine in Pediatric out-of-Hospital Cardiopulmonary Arrest**. *Pediatrics* 1995, **95**(6):901.
8. Glajchen M, Magen R: **Evaluating process, outcome, and satisfaction in community-based cancer support groups**. *Support Groups: Current Perspective on Theory and Practice* 1995, **18**:27-40.
9. Granger CE, George C, Shelly MP: **The management of bereavement on intensive care units**. *Intensive Care Medicine* 1995, **21**(5):429-436.
10. Heiney SP, Ruffin J, Goon-Johnson K: **The effects of a support group on selected psychosocial outcomes of bereaved parents whose child died from cancer**. *Journal of Pediatric Oncology Nursing* 1995, **12**(2):51-58; discussion 59-61.
11. Jensen BJA: **Caregiver responses to a theoretically based intervention program: case study analysis**. *Ph.d.*: THE UNIVERSITY OF TEXAS AT AUSTIN; 1995.
12. Klockevold B: **Multi-media: herbal videos**. *American Herb Association Quarterly Newsletter* 1995, **11**(3):13.
13. Reichenberg-Ullman J: **The case of the dream warrior**. *Small Remedies & Interesting Cases: Professional Case Conferences* 1995, **2**:271-282.
14. Robinson JH: **Grief responses, coping processes, and social support of widows: research with Roy's model**. *Nursing Science Quarterly* 1995, **8**(4):158-164.
15. Schnyder U: **[Family-based crisis intervention after attempted suicide]**. *Nervenarzt* 1995, **66**(7):554-560.
16. Schreiner-Engel P, Walther Virginia N, Mindes J, Lynch L, Berkowitz Richard L: **First-trimester multifetal pregnancy reduction: Acute and persistent psychologic reactions**. *American Journal of Obstetrics & Gynecology* 1995, **172**(2 PART 1):541-547.
17. Sikkema KJ, Kalichman SC, Kelly JA, Koob JJ: **Group intervention to improve coping with AIDS-related bereavement: model development and an illustrative clinical example**. *AIDS Care* 1995, **7**(4):463-475.
18. Tarockova T: **Grief Counseling as an Emerging Sphere of Psychological Counseling**. *Cesk Psychol* 1995, **39**(3):229.

19. Thayre K: **Never going to be easy: giving bad news**. *Nursing Standard* 1995, **9**(50):RCN Nurs Update: 3-12.
20. Tudiver F, Permaul-Woods J, Hilditch J, Harmina J, Saini S: **Do widowers use the health care system differently? Does intervention make a difference?** *Canadian Family Physician* 1995, **41**:392-400.

1996 (18)

1. Brown KLG: **Grief as a basic conditioning factor affecting the self-care agency and self-care of family caregivers of persons with neurotrauma**. *Ph.d.*: WAYNE STATE UNIVERSITY; 1996.
2. Della Selva PC: **Intensive short-term dynamic psychotherapy: Theory and technique**; 1996.
3. Erwin KT: **Group techniques for aging adults: Putting geriatric skills enhancement into practice**; 1996.
4. Forte JA, Barrett AV, Campbell MH: **Patterns of social connectedness and shared work: a symbolic interactionist perspective**. *Social Work with Groups* 1996, **19**(1):29-51.
5. Garcia-Garcia JA, Landa Petralanda V, Trigueros Manzano MC, Calvo Aedo P, Gaminde Inda I: **Grief for loss of a spouse: a study with discussion groups in primary care**. *Atencion Primaria* 1996, **18**(9):475-479.
6. Green J: **Mothers in "Incest Families": A Critique of Blame and Its Destructive Sequels**. *Violence Against Women* 1996, **2**(3):322-348.
7. Krongrad A, Lai H, Burke MA, Goodkin K, Lai SH: **Marriage and mortality in prostate cancer**. *J Urol* 1996, **156**(5):1696.
8. Leavitt KS, Morrison JA, Gardner SA, Gallagher MM: **Group play therapy for cumulatively traumatized child survivors of familial AIDS**. *International Journal of Play Therapy* 1996, **5**(1):1-17.
9. Lee C, Slade P: **Miscarriage as a traumatic event: a review of the literature and new implications for intervention**. *Journal of Psychosomatic Research* 1996, **40**(3):235-244.
10. Lev EL, Owen SV: **A measure of self-care self-efficacy... Strategies Used by People to Promote Health (SUPPH)**. *Research in Nursing & Health* 1996, **19**(5):421-429.
11. Light L: **Healing with homeopathy**. *Vegetarian Times* 1996, **Oct**(230):104-106.
12. McCreight BH: **A comparative study of two types of group therapy for adult female survivors of childhood sexual abuse**. *Dissertation Abstracts International: Section B: the Sciences & Engineering* 1996, **56**(11-B):6399.
13. Moore K: **Effects of a brief task-focused intervention on the bereavement outcome of mid-life widows**. *Dissertation Abstracts International* 1996, **56**(11-A):4283.
14. Mundt C: **Psychotherapy of depression: The theoretical background and its practical consequences**. *Nervenarzt* 1996, **67**(3):183.
15. Murphy SA, Baugher R, Lohan J, Scheideman J, Heerwagen J, Johnson LC, Tillery L, Grover MC: **Parents' evaluation of a preventive intervention**

following the sudden, violent deaths of their children. *Death Studies* 1996, **20**(5):453-468.
16. Nielsen S HM, Moller A, Granberg S: **Bereavement, grieving and psychological morbidity after first trimester spontaneous abortion: comparing expectant management with surgical evacuation**. *Human Reproduction* 1996, **11**(8):1767-1770.
17. Pohle MS: **Battered women: Self-definition**. *Dissertation Abstracts International: Section B: the Sciences & Engineering* 1996, **57**(5-B):3418.
18. Tonkins S, Lambert M: **A treatment outcome study of bereavement groups for children**. *Child and Adolescent Social Work Journal* 1996, **13**(1):3-21.

1997 (21)

1. Anonymous: **Natural killer cells need sleep**. *Oriental Medicine Journal* 1997, **6**(3/4):8-9.
2. Anthony KH: **Bitter Homes and Gardens: The Meanings of Home to Families of Divorce**. *Journal of Architectural & Planning Research* 1997, **14**(1):1-19.
3. Beale MQ: **A study of the function and meaning of imagery experienced by rape survivors**. *Dissertation Abstracts International* 1997, **57**(8-A):3678.
4. Eick SE: **A bereavement support group for family hospice of Indiana County**. *Dissertation Abstracts International: Section B: the Sciences & Engineering* 1997, **58**(1-B):0415.
5. Frank E, Prigerson Holly G, Shear MK, Reynolds Charles F, III: **Phenomenology and treatment of bereavement-related distress in the elderly**. *International Clinical Psychopharmacology* 1997, **12**(SUPPL. 7):S25-S29.
6. Kavanaugh K: **Parents' experience surrounding the death of a newborn whose birth is at the margin of viability**. *JOGNN - Journal of Obstetric, Gynecologic, & Neonatal Nursing* 1997, **26**(1):43-51.
7. Kissane DW, McKenzie DP, Bloch S: **Family coping and bereavement outcome**. *Palliative Medicine* 1997, **11**(3):191-201.
8. Kissane DW, Bloch S, Miach P, Smith GC, Seddon A, Keks N: **Cognitive-existential group therapy for patients with primary breast cancer - Techniques and themes**. *Psycho-Oncology* 1997, **6**(1):25.
9. McKibbin CL, Guarnaccia CA, Hayslip B, Jr., Murdock ME: **Locus of control perceptions among conjugally bereaved older adults: a pilot study**. *International Journal of Aging & Human Development* 1997, **44**(1):37-45.
10. Murphy SA: **A bereavement intervention for parents following the sudden, violent deaths of their 12-28-year-old children: description and applications to clinical practice**. *Canadian Journal of Nursing Research* 1997, **29**(4):51-72.
11. Murphy K, Hanrahan P, Luchins D: **A survey of grief and bereavement in nursing homes: the importance of hospice grief and bereavement for the end-stage Alzheimer's disease patient and family.[comment]**. *Journal of the American Geriatrics Society* 1997, **45**(9):1104-1107.

12. Neugebauer R, Kline J, Shrout P, Skodol A, Oconnor P, Geller PA, Stein Z, Susser M: **Major depressive disorder in the 6 months after miscarriage**. *JAMA-J Am Med Assoc* 1997, **277**(5):383.
13. Oates WE: **Reconciling with unfulfilled dreams at the end of life**. In: *The aging family: New visions in theory, practice, and reality (pp 259-269) xi.* Edited by Hargrave TD, Hanna SM; 1997: 321.
14. Prince MJ, Harwood RH, Blizard RA, Thomas A, Mann AH: **Social support deficits, loneliness and life events as risk factors for depression in old age. The Gospel Oak Object .6**. *Psychological Medicine* 1997, **27**(2):323.
15. Rashotte J, Fothergill-Bourbonnais F, Chamberlain M: **Pediatric intensive care nurses and their grief experiences: a phenomenological study**. *Heart & Lung: Journal of Acute & Critical Care* 1997, **26**(5):372-386.
16. Reviews NHSCf, Dissemination: **Patient and carer preference for, and satisfaction with, specialist models of palliative care**. *Evidence Based Medicine Jul Aug* 1997, **2**:123.
17. Schaap AH, Wolf H, Bruinse HW, Barkhof-van de Lande S, Treffers PE: **Long-term impact of perinatal bereavement. Comparison of grief reactions after intrauterine versus neonatal death**. *European Journal of Obstetrics, Gynecology, & Reproductive Biology* 1997, **75**(2):161-167.
18. Sowden AJ, Tilford S, Delaney F, Vogels M, Gilbody S, Sheldon TA: **Mental health promotion in high risk groups**. *Qual Health Care* 1997, **6**(4):219.
19. Van Epps J, Opie ND, Goodwin T: **Themes in the bereavement experience of inner city adolescents**. *Journal of Child & Adolescent Psychiatric Nursing* 1997, **10**(1):25-36.
20. Wolchik SA, Sandler IN (eds.): **Handbook of children's coping: Linking theory and intervention**; 1997.
21. Wolfson L, Miller M, Houck P, Ehrenpreis L, Stack JA, Frank E, Cornes C, Mazumdar S, Kupfer DJ, Reynolds CF: **Foci of interpersonal psychotherapy (IPT) in depressed elders: Clinical and outcome correlates in a combined IPT/nortriptyline protocol**. *Psychother Res* 1997, **7**(1):45.

1998 (30)

1. Addington-Hall J WLJCKSMM: **A randomised controlled trial of postal versus interviewer administration of a questionnaire measuring satisfaction with, and use of, services received in the year before death**. *Journal of Epidemiology & Community Health* 1998, **52**(12):802-807.
2. Balk DE, Lampe S, Sharpe B, Schwinn S, Holen K, Cook L, Dubois R, 3rd: **TAT results in a longitudinal study of bereaved college students**. *Death Studies* 1998, **22**(1):3-21.
3. Bonanno GA NC, Gunzerath L, Keltner D, Horowitz MJ: **Interpersonal ambivalence, perceived relationship adjustment, and conjugal loss**. *Journal of Consulting & Clinical Psychology* 1998, **66**(6):1012-1022.
4. Burkhalter JE: **A bereavement support group intervention: Effects on bereavement-specific situational coping and associations of dispositional coping style and situational coping with psychological distress and immune function in bereaved hiv seronegative and seropositive gay men**.

Dissertation Abstracts International: Section B: the Sciences & Engineering 1998, **59**(2-B):0865.

5. Daaleman TP, Frey B: **Prevalence and patterns of physician referral to clergy and pastoral care providers**. *Archives of Family Medicine* 1998, **7**(6):548-553.
6. Goodkin K, Feaster Daniel J, Asthana D, Blaney Nancy T, Kumar M, Baldewicz T, Tuttle Raymond S, Maher Kevin J, Baum Marianna K, Shapshak P *et al*: **A bereavement support group intervention is longitudinally associated with salutary effects on the CD4 cell count and number of physician visits**. *Clinical & Diagnostic Laboratory Immunology* 1998, **5**(3):382-391.
7. Gould D: **Grief after pregnancy loss was predicted by length of pregnancy, neuroticism, psychiatric symptoms, and absence of other children [commentary on Janssen HJ, Cuisinier MC, de Graauw KP, et al. A prospective study of risk factors predicting grief intensity following pregnancy loss. ARCH GEN PSYCHIATRY 1997 Jan;54:56-61]**. *Evidence-Based Nursing* 1998, **1**(2):54.
8. Hunter S, Grimmer M, Wayne B, Carter H: **Palliative care in Forth Valley: perspective of community nursing providers on service and education needs**. *Health Bulletin* 1998, **56**(1):511-517.
9. Jones BL: **Integrating hearing loss was a complex and dynamic process for patients [commentary on Herth K. Integrating hearing loss into one's life. QUAL HEALTH RES 1998;8(2):207-23]**. *Evidence-Based Nursing* 1998, **1**(4):131.
10. Kauffmann E: **Parents experienced cycles of defining and managing adversity in caring for a child with a chronic progressive illness [commentary on Gravelle AM. Caring for a child with a progressive illness during the complex chronic phase: parents' experience of facing adversity. J ADV NURS 1997;25(4):738-45]**. *Evidence-Based Nursing* 1998, **1**(1):28.
11. Lasser R, Siegel E, Dukoff R, Sunderland T: **Diagnosis and treatment of geriatric depression**. *CNS Drugs* 1998, **9**(1):17.
12. Liebert DT: **The married widow: A correlational study of grief and social support networks amongst non-residential spouses of long-term care nursing home residents**. *Dissertation Abstracts International: Section B: the Sciences & Engineering* 1998, **59**(2-B):0598.
13. Lloyd-Williams M, Wilkinson C, Lloyd-Williams FF: **Do bereaved children consult the primary health care team more frequently?** *European Journal of Cancer Care* 1998, **7**(2):120-124.
14. Lohan JA: **Parents' perceptions of family functioning and sibling grief in families who have experienced the violent death of an adolescent or young adult child**. *Dissertation Abstracts International: Section B: the Sciences & Engineering* 1998, **59**(6-B):2682.
15. Luscombe G, Brodaty H, Freeth S: **Younger people with dementia: diagnostic issues, effects on carers and use of services**. *International Journal of Geriatric Psychiatry* 1998, **13**(5):323-330.
16. March JS, Amaya-Jackson L, Murray MC, Schulte A: **Cognitive-behavioral psychotherapy for children and adolescents with posttraumatic stress**

disorder after a single-incident stressor. *J Am Acad Child Adolesc Psychiatr* 1998, **37**(6):585.
17. Metcalf L: **Solution focused group therapy: Ideas for groups in private practice, schools, agencies, and treatment programs**; 1998.
18. Miller MD, Reynolds CF: **Nortriptyline for Grief-Related Depression in Elders**. *151st Annual Meeting of the American Psychiatric Association Toronto, Ontario, Canada 30th May 4th June* 1998(No).
19. Mufson L, Moreau D: **Interpersonal psychotherapy for adolescent depression**. In: *Interpersonal psychotherapy review of psychiatry series (pp 35-66) xv.* Edited by Markowitz JC; 1998: 162.
20. Murphy SA, Johnson C, Cain KC, Das Gupta A, Dimond M, Lohan J, Baugher R: **Broad-spectrum group treatment for parents bereaved by the violent deaths of their 12- to 28-year-old children: a randomized controlled trial**. *Death Studies* 1998, **22**(3):209-235.
21. Pelletier-Hibbert M: **Coping strategies used by nurses to deal with the care of organ donors and their families**. *Heart Lung* 1998, **27**(4):230.
22. Reynolds NR, Alonzo AA: **HIV informal caregiving: emergent conflict and growth**. *Research in Nursing & Health* 1998, **21**(3):251-260.
23. Robinson SM, Mackenzie-Ross S, Campbell Hewson GL, Egleston CV, Prevost AT: **Psychological effect of witnessed resuscitation on bereaved relatives**. *Lancet (North American Edition)* 1998, **352**(9128):614-617.
24. Schlernitzauer M BAJGMDPHGSJAMMDPRE, Reynolds CF, 3rd: **Recruitment methods for intervention research in bereavement-related depression. Five years' experience**. *American Journal of Geriatric Psychiatry* 1998, **6**(1):67-74.
25. Sherman JJ: **Effects of psychotherapeutic treatments for PTSD: A meta-analysis of controlled clinical trials**. *Journal of Traumatic Stress* 1998, **11**(3):413.
26. Snowdon C, Garcia J, Elbourne D: **Reactions of participants to the results of a randomised controlled trial: exploratory study.[comment]**. *BMJ* 1998, **317**(7150):21-26.
27. Strydom H, Fourie J: **The Content and Evaluation of a Bereavement Counselling Programme for Parents after the Death of a Child**. *Maatskaplike Werk/Social Work* 1998, **34**(4):389-405.
28. Tanner JL, Dechert MP, Frieden IJ: **Growing up with a facial hemangioma: parent and child coping and adaptation**. *Pediatrics* 1998, **101**(3 Pt 1):446-452.
29. Thomas SB, Leite B, Duncan T: **Breaking the Cycle of Violence among Youth Living in Metropolitan Atlanta: A Case History of Kids Alive and Loved**. *Health Education & Behavior* 1998, **25**(2):160-174.
30. Zygmont M, Prigerson HG, Houck PR, Miller MD, Shear MK, Jacobs S, Reynolds CFI: **A post hoc comparison of paroxetine and nortriptyline for symptoms of traumatic grief**. *Journal of Clinical Psychiatry* 1998, **59**(5):241-245.

1999 (26)

1. Anonymous: **The Upledger Foundation update on current research projects**. *Connections Magazine* 1999, **Autumn**(36):4.
2. Beutel M, Kupfer J, Kirchmeyer P, Kehde S, Kohn FM, Schroeder-Printzen I, Gips H, Herrero HJG, Weidner W: **Treatment-related stresses and depression in couples undergoing assisted reproductive treatment by IVF or ICSI**. *Andrologia* 1999, **31**(1):27.
3. Blok GA, van Dalen J, Jager KJ, Ryan M, Wijnen RMH, Wight C, Morton JM, Morley M, Cohen B: **The European Donor Hospital Education Programme (EDHEP): addressing the training needs of doctors and nurses who break bad news, care for the bereaved, and request donation**. *Transplant International* 1999, **12**(3):161.
4. Cohen I, van Lunsen RH: **Abortion service in Amsterdam: deciding and coping in a liberal system**. *European Journal of Contraception & Reproductive Health Care* 1999, **4**(1):1-6.
5. Coleman GL: **Eye movement desensitization and reprocessing in the treatment of posttraumatic stress disorder: An investigational study of the eye movement component using a within-subject design**. *Dissertation Abstracts International: Section B: the Sciences & Engineering* 1999, **60**(4-B):1846.
6. Dent A: **Evaluation of community interventions for bereaved parents using a randomised controlled trial**. *National Research Register* 1999.
7. Dominguez ME: **The treatment of complicated mourning in children**. *Dissertation Abstracts International: Section B: the Sciences & Engineering* 1999, **60**(6-B):2938.
8. Dowdney L, Wilson R, Maughan B, Allerton M, Schofield P, Skuse D: **Psychological disturbance and service provision in parentally bereaved children: prospective case-control study**. *BMJ* 1999, **319**(7206):354-357.
9. Engler AJ: **Grief, physical symptoms, and decision-making in mothers of newborns who die. (bereavement, newborn death, coping)**. *Dissertation Abstracts International: Section B: the Sciences & Engineering* 1999, **60**(4-B):1528.
10. Gess JK: **A Community Wide Study of the Hospice Referral Process**. *Dissertation Abstracts International* 1999, **60**(6):2231-A.
11. Goodkin K, Blaney NT, Feaster DJ, Baldewicz T, Burkhalter JE, Leeds B: **A randomized controlled clinical trial of a bereavement support group intervention in human immunodeficiency virus type 1-seropositive and -seronegative homosexual men**. *Archives of General Psychiatry* 1999, **56**(1):52-59.
12. Gray J, Hedge B: **Psychological distress and coping in the partners of gay men with HIV-related disease**. *Br J Health Psychol* 1999, **4**:117.
13. Grinwis AA: **Loss of the lifelong partner: Implications for social adaptation**. *Dissertation Abstracts International* 1999, **59**(12-A):4498.
14. Huss SN, Ritchie M: **Effectiveness of a group for parentally bereaved children**. *Journal for Specialists in Group Work* 1999, **24**(2):186-196.

15. Kasprzak CJ: **Multisystemic theory of post adoption services and treatment: A program design**. *Dissertation Abstracts International: Section B: the Sciences & Engineering* 1999, **60**(3-B):1303.
16. Kendrick T: **Primary care options to prevent mental illness**. *Ann Med* 1999, **31**(6):359.
17. Maddocks I: **Book reviews**. *Palliative Medicine* 1999, **13**(2):177-179.
18. Nelligan P: **A bereavement support group reduced grief and distress in bereaved, homosexual men [commentary on Goodkin K, Blaney NT, Feaster D, et al. A randomized controlled clinical trial of a bereavement support group intervention in human immunodeficiency virus type 1-seropositive and -seronegative homosexual men. ARCH GEN PSYCHIATRY 1999 Jan;56:52-9]**. *Evidence-Based Nursing* 1999, **2**(4):116.
19. Reynolds C, 3rd, Miller MD, Pasternak RE, Frank E, Perel JM, Cornes C, Houck PR, Mazumdar S, Dew MA, Kupfer DJ: **Treatment of bereavement-related major depressive episodes in later life: a controlled study of acute and continuation treatment with nortriptyline and interpersonal psychotherapy**. *American Journal of Psychiatry* 1999, **156**(2):202-208.
20. Saunderson EM, Ridsdale L: **General practitioners' beliefs and attitudes about how to respond to death and bereavement: qualitative study.[comment]**. *BMJ* 1999, **319**(7205):293-296.
21. Segal D, Bogaards J, Becker L, Chatman C: **Effects of emotional expression on adjustment to spousal loss among older adults**. *Journal of MENT HEALTH AGING* 1999, **5**(4):297-310.
22. Spruyt O: **Community-based palliative care for Bangladeshi patients in east London. Accounts of bereaved carers**. *Palliative Medicine* 1999, **13**(2):119-129.
23. Swanson KM: **Research-based practice with women who have had miscarriages**. *Image - the Journal of Nursing Scholarship* 1999, **31**(4):339-345.
24. Swanson KM: **Effects of caring, measurement, and time on miscarriage impact and women's well-being**. *Nurs Res* 1999, **48**(6):288.
25. Taylor M, Reynolds C, 3rd , Frank E, Dew M, Mazumdar S, Houck P, Kupfer D: **EEG sleep measures in later-life bereavement depression. A randomized, double-blind, placebo-controlled evaluation of nortriptyline**. *American Journal of Geriatric Psychiatry* 1999, **7**(1):41-47.
26. Yates P, Stetz KM: **Families' awareness of and response to dying**. *Oncology Nursing Forum* 1999, **26**(1):113-120.

2000 (23)

1. Adams C, Bonnett B, Meek A: **Predictors of owner response to companion animal death in 177 clients from 14 practices in Ontario**. *Journal of the American Veterinary Medical Association* 2000, **217**(9):1303-1309.
2. Carver EA: **Depression and loss: The effects of a short term bereavement support group for children and families**. *Dissertation Abstracts International: Section B: the Sciences & Engineering* 2000, **60**(11-B):5765.

3. Chambers HM, Chan FY: **Support for women/families after perinatal death**. *Cochrane Database of Systematic Reviews* 2000(2):CD000452.
4. Cote-Arsenault D, Marshall R: **One foot in -- one foot out: weathering the storm of pregnancy after perinatal loss**. *Research in Nursing & Health* 2000, **23**(6):473-485.
5. Coulthard P, Bridgman CM, Larkin A, Worthington HV: **Appropriateness of a Resuscitation Council (UK) advanced life support course for primary care dentists**. *British Dental Journal* 2000, **188**(9):507-512.
6. Cuthbertson SJ, Margetts MA, Streat SJ: **Bereavement follow-up after critical illness**. *Critical Care Medicine* 2000, **28**(4):1196.
7. Donnelly JM, Kornblith AB, Fleishman S, Zuckerman E, Raptis G, Hudis CA, Hamilton N, Payne D, Massie MJ, Norton L *et al*: **A pilot study of interpersonal psychotherapy by telephone with cancer patients and their partners**. *Psycho-Oncology* 2000, **9**(1):44-56.
8. Durland SE: **Adult bereavement: A critical review of theories and treatment outcome studies**. *Dissertation Abstracts International: Section B: the Sciences & Engineering* 2000, **60**(9-B):4885.
9. Esplen MJTBHJGGLANSSNBK, and Field B: **A supportive-expressive group intervention for women with a family history of breast cancer: results of a phase ii study**. *Psycho Oncology* 2000, **9**(3):243-252.
10. Fogarty JA: **The magical thoughts of grieving children: Treating children with complicated mourning and advice for parents**; 2000.
11. Forchuk C: **Dealing with a family member who has a mental illness was a long term, frustrating, and confusing process before acceptance occurred... commentary on Karp DA, Tanarugsachock V. Mental illness, caregiving, and emotion management. QUAL HEALTH RES 2000 Jan;10:6-25**. *Evidence-Based Nursing* 2000, **3**(4):133.
12. Fortner BV: **The effectiveness of grief counseling and therapy: A quantitative review**. *Dissertation Abstracts International: Section B: the Sciences & Engineering* 2000, **60**(8-B):4221.
13. Holst-Warhaft G: **The cue for passion: Grief and its political uses**; 2000.
14. Jacobs S, Prigerson H: **Psychotherapy of traumatic grief: a review of evidence for psychotherapeutic treatments**. *Death Studies* 2000, **24**(6):479-495.
15. Johnson BS: **Mothers' perceptions of parenting children with disabilities**. *MCN, American Journal of Maternal Child Nursing* 2000, **25**(3):127-132.
16. Kaunonen M, Tarkka M, Laippala P, Paunonen-Ilmonen M: **The impact of supportive telephone call intervention on grief after the death of a family member**. *Cancer Nursing* 2000, **23**(6):483-491.
17. Loy MJ: **A study of the effectiveness of a camp intervention for bereaved adolescents. (counseling, grief intervention)**. *Dissertation Abstracts International* 2000, **60**(7-A):2386.
18. McRae S, Caty S, Nelder M, Picard L: **Palliative care on Manitoulin Island. Views of family caregivers in remote communities**. *Canadian Family Physician* 2000, **46**:1301-1307.

19. Neimeyer RA: **Searching for the meaning of meaning: grief therapy and the process of reconstruction.[comment]**. *Death Studies* 2000, **24**(6):541-558.
20. Partnership for Caring I: **On the shelves**. *VOICES: The newsletter of Partnership for Caring* 2000, **1**(3):8.
21. Patterson PR: **Living with grief after pregnancy loss: Perspectives of African American women**. *Ph.D.*: University of California, San Francisco; 2000.
22. Rich DE: **The impact of postpregnancy loss services on grief outcome: Integrating research and practice in the design of perinatal bereavement programs**. *Illness, Crisis & Loss* 2000, **8**(3):244-264.
23. Schreiber H: **Parents experiencing a perinatal death found that their interactions with social institutions devalued their loss [commentary on Malacrida C. Complicating mourning: the social economy of perinatal death. QUAL HEALTH RES 1999 Jul;9(4):504-19]**. *Evidence-Based Nursing* 2000, **3**(1):27.

2001 (36)

1. Abe N, Catlin A, Mihara D: **End of life in the NICU. A study of ventilator withdrawal**. *MCN, American Journal of Maternal Child Nursing* 2001, **26**(3):141-146.
2. Armstrong D: **Exploring fathers' experiences of pregnancy after a prior perinatal loss**. *MCN, American Journal of Maternal Child Nursing* 2001, **26**(3):147-153.
3. Campbell BB: **Shattered futures, mended lives: The ritualized mourning of mothers of stillborn babies**. *Dissertation Abstracts International* 2001, **61**(10-A):3825.
4. Colsen TL: **Fathers and perinatal loss: Their conscious and unconscious experiences**. *Dissertation Abstracts International: Section B: the Sciences & Engineering* 2001, **62**(4-B):2051.
5. Curtis K, Newman T: **Do community-based support services benefit bereaved children? A review of empirical evidence**. *Child: Care, Health & Development* 2001, **27**(6):487-495.
6. DiMarco MA, Menke EM, McNamara T: **Evaluating a support group for perinatal loss**. *MCN, American Journal of Maternal Child Nursing* 2001, **26**(3):135-140.
7. Edwards WT, Bylund RA, Wheeler AL: **Race and Complicated Grief among Male Inmates**. In.: Southern Sociological Society; 2001.
8. Fry PS: **Predictors of health-related quality of life perspectives, self-esteem, and life satisfactions of older adults following spousal loss: an 18-month follow-up study of widows and widowers**. *Gerontologist* 2001, **41**(6):787-798.
9. Fry PS: **The unique contribution of key existential factors to the prediction of psychological well-being of older adults following spousal loss**. *Gerontologist* 2001, **41**(1):69-81.

10. Gonzalez Villar M, Madoz Zubillaga MN, Calle Irastorza F, Jurio Burgui JJ, Arillo Crespo A, Fuertes Goni MC: **Support intervention in grieving patients**. *Atencion Primaria* 2001, **27**(2):101-107.
11. Goodkin K, Baldewicz TT, Asthana D, Khamis I, Blaney NT, Kumar M, Burkhalter JE, Leeds B, Shapshak P: **A bereavement support group intervention affects plasma burden of human immunodeficiency virus type 1:report of a randomized controlled trial**. *Journal of Human Virology* 2001, **4**(1):44-54.
12. Guthrie SJ, Hill KM, Muers MF: **Living with severe COPD. A qualitative exploration of the experience of patients in Leeds**. *Respir Med* 2001, **95**(3):196.
13. Hatton R: **Helping people cope with homicide bereavement**. *Dissertation Abstracts International: Section B: the Sciences & Engineering* 2001(10-B):5565.
14. Hilliard RE: **The effects of music therapy-based bereavement groups on mood and behavior of grieving children: a pilot study**. *Journal of Music Therapy* 2001, **38**(4):291-306.
15. Kravetz BE: **Accreditation standards for mental healthcare professionals in the treatment of HIV-positive women with children: A program design (immune deficiency)**. *Dissertation Abstracts International: Section B: the Sciences & Engineering* 2001, **61**(8-B):4411.
16. Laakso H, Paunonen-Ilmonen M: **Mothers' grief following the death of a child**. *Journal of Advanced Nursing* 2001, **36**(1):69-77.
17. Lange A, van de Ven JP, Schrieken B, Emmelkamp PM: **Interapy, treatment of posttraumatic stress through the Internet: a controlled trial**. *Journal of Behavior Therapy & Experimental Psychiatry* 2001, **32**(2):73-90.
18. Links P: **Therapists of patients who committed suicide reported a wide range of emotional responses**. *Evidence-Based Mental Health* 2001, **4**(3):96.
19. Massimo L: **[Home care services and the role of "caregivers"]**. *Minerva Pediatrica* 2001, **53**(3):161-169.
20. Meuser TM, Marwit SJ: **A comprehensive, stage-sensitive model of grief in dementia caregiving**. *Gerontologist* 2001, **41**(5):658-670.
21. Oliver RC, Sturtevant JP, Scheetz JP, Fallat ME: **Beneficial effects of a hospital bereavement intervention program after traumatic childhood death**. *Journal of Trauma-Injury Infection & Critical Care* 2001, **50**(3):440-446; discussion 447-448.
22. Partnership for Caring I: **Visit our new website**. *VOICES: The newsletter of Partnership for Caring* 2001, **2**(1):8.
23. Piper W, McCallum M, Joyce A, Rosie J, Ogrodniczuk J: **Patient personality and time-limited group psychotherapy for complicated grief**. *International Journal of Group Psychotherapy* 2001, **51**(4):525-552.
24. Planavsky LA, Mion LC, Litaker DG, Kippes CM, Mehta N: **Ending a nurse practitioner-patient relationship: uncovering patients' perceptions**. *Journal of the American Academy of Nurse Practitioners* 2001, **13**(9):428-432.
25. Prigerson HG, Jacobs SC: **Perspectives on care at the close of life. Caring for bereaved patients: "all the doctors just suddenly go"**. *JAMA* 2001, **286**(11):1369-1376.

26. Rillstone P, Hutchinson SA: **Managing the reemergence of anguish: pregnancy after a loss due to anomalies**. *JOGNN - Journal of Obstetric, Gynecologic, & Neonatal Nursing* 2001, **30**(3):291-298.
27. Ringdal GI, Jordhoy MS, Ringdal K, Kaasa S: **The first year of grief and bereavement in close family members to individuals who have died of cancer**. *Palliative Medicine* 2001, **15**(2):91-105.
28. Ringdal GI, Jordhoy MS, Ringdal K, Kaasa S: **Factors affecting grief reactions in close family members to individuals who have died of cancer**. *J Pain Symptom Manage* 2001, **22**(6):1016.
29. Sakai C, Paperny D, Mathews M, Tanida G, Boyd G, Simons A, Yamamoto C, Mau C, Nutter L: **Thought Field Therapy clinical applications: utilization in an HMO in behavioral medicine and behavioral health services.[comment]**. *Journal of Clinical Psychology* 2001, **57**(10):1215-1227.
30. Salloum A, Avery L, McClain RP: **Group psychotherapy for adolescent survivors of homicide victims: a pilot study**. *Journal of the American Academy of Child & Adolescent Psychiatry* 2001, **40**(11):1261-1267.
31. Sanchez NA: **Mothers' perceptions of benefits of perinatal loss support offered at a major university hospital**. *Journal of Perinatal Education* 2001, **10**(2):23-30.
32. Schneider KJ, Bugental JFT, Pierson JF (eds.): **The handbook of humanistic psychology: Leading edges in theory, research, and practice**; 2001.
33. Stewart M, Craig D, MacPherson K, Alexander S: **Promoting positive affect and diminishing loneliness of widowed seniors through a support intervention**. *Public Health Nursing* 2001, **18**(1):54-63.
34. Warner J, Metcalfe C, King M: **Evaluating the use of benzodiazepines following recent bereavement**. *British Journal of Psychiatry* 2001, **178**(1):36-41.
35. Wilson RE: **Parents' support of their other children after a miscarriage or perinatal death**. *Early Human Development* 2001, **61**(2):55-65.
36. Zisook S, Schuchter S, Pedrelli P, Sable J, Deaciuc S: **Bupropion sustained release for bereavement: Results of an open trial**. *Journal of Clinical Psychiatry* 2001, **64**(2):227-230.

2002 (18)

1. Birtwistle J, Payne S, Smith P, Kendrick T: **The role of the district nurse in bereavement support**. *Journal of Advanced Nursing* 2002, **38**(5):467-478.
2. Campbell FR: **The influence of an active postvention on the length of time elapsed before survivors of suicide seek treatment**. *Dissertation Abstracts International* 2002, **63**(2-A):753.
3. Cannold L: **Understanding and responding to anti-choice women-centred strategies**. *Reproductive Health Matters* 2002, **10**(19):171-179.
4. Consedine NS, Magai C, Cohen CI, Gillespie M: **Ethnic variation in the impact of negative affect and emotion inhibition on the health of older adults**. *J Gerontol Ser B-Psychol Sci Soc Sci* 2002, **57**(5):P396.

5. Dent A: **Family support after sudden child death**. *Community Practitioner* 2002, **75**(12):469-473.
6. Harkness KL, Shear MK, Frank E, Silberman RA: **Traumatic grief treatment: case histories of 4 patients**. *Journal of Clinical Psychiatry* 2002, **63**(12):1113-1120.
7. Hersen M, Sledge W (eds.): **Encyclopedia of psychotherapy (Vol. 1: A-H)**. San Diego, CA: Academic Press.; 2002.
8. Higgins MP: **Parental bereavement and religious factors**. *Omega-J Death Dying* 2002, **45**(2):187.
9. Kitson C: **Fathers experienced stillbirth as a waste of life and needed to protect their partners and express grief in their own way**. *Evidence-Based Nursing* 2002, **5**(2):61.
10. Laumann GM: **The impact of a school-based intervention program on the divorce adjustment of latency-aged children: An evaluation of the Rainbows program**. *Dissertation Abstracts International: Section B: the Sciences & Engineering* 2002, **63**(2-B):1066.
11. Locicero JP: **A comparison of non-certified and certified grief counselors in regard to education, experience, credentials, and supervision**. *Omega-J Death Dying* 2002, **46**(1):5.
12. Marwit SJ, Meuser TM: **Development and initial validation of an inventory to assess grief in caregivers of persons with Alzheimer's disease**. *Gerontologist* 2002, **42**(6):751-765.
13. Mitchell GK: **How well do general practitioners deliver palliative care? A systematic review**. *Palliative Medicine* 2002, **16**(6):457.
14. Rowa-Dewar N: **Do interventions make a difference to bereaved parents? A systematic review of controlled studies**. *International Journal of Palliative Nursing* 2002, **8**(9):452-457.
15. Solomon RM: **Treatment of violated assumptive worlds with EMDR**. In: *Loss of the assumptive world: A theory of traumatic loss The series in trauma and loss.* Edited by Kauffman J. New York, NY: Brunner-Routledge; 2002: 117-126.
16. Stoddart K, Burke L, Temple V: **Outcome evaluation of bereavement groups for adults with intellectual disabilities**. *Journal of Applied Research in Intellectual Disabilities* 2002, **15**:28-35.
17. Utz RL, Carr D, Nesse R, Wortman CB: **The effect of Widowhood on older adults' social participation: Aan evaluation of activity, disengagement, and continuity theories**. *Gerontologist* 2002, **42**(4):522.
18. Wayment HA, Vierthaler J: **Attachment style and bereavement reactions**. *J Loss Trauma* 2002, **7**(2):129.

2003 (27)

1. Ahmed N, Ahmedzai S, Vora V, Hillam S, Paz S: *Cochrane Database of Systematic Reviews* 2003, **1**:1.
2. Barlow J, Coren E: *Cochrane Database of Systematic Reviews* 2003, **1**:1.
3. Bower P, Rowland N, Mellor Clark J, Heywood P, Godfrey C, Hardy R: *Cochrane Database of Systematic Reviews* 2003, **1**:1.

4. Chambers HM, Chan FY: **Support for women/families after perinatal death**. In. Oxford: The Cochrane Library; 2003.
5. Churchill R: **Enhanced primary care may encourage remission in depression**. *Evidence-Based Mental Health* 2003, **6**(2):47.
6. Faraclas H-I: **A position paper on the nature of the counseling relationship within the philosophy of neuropathic medicine**. *Townsend Letter for Doctors & Patients* 2003, **Feb/Mar**(235/236):64-66.
7. Foster J, Cole M: *Cochrane Database of Systematic Reviews* 2003, **1**:1.
8. Geron Y, Ginzburg K, Solomon Z: **Predictors of bereaved parents' satisfaction with group support: An Israeli perspective**. *Death Studies* 2003, **27**(5):405.
9. Howarth GR, Alfirevic Z: *Cochrane Database of Systematic Reviews* 2003, **1**:1.
10. Ismail K, McGuire H, Winkley K: *Cochrane Database of Systematic Reviews* 2003, **1**:1.
11. Jefferson TO, Tyrrell D: *Cochrane Database of Systematic Reviews* 2003, **1**:1.
12. Joy CB, Adams CE, Rice K: *Cochrane Database of Systematic Reviews* 2003, **1**:1.
13. Lachs MS, Boyer P: **Timing the talk**. *Prevention* 2003, **55**(1):154-156.
14. Lumley J, Watson L, Watson M, Bower C: *Cochrane Database of Systematic Reviews* 2003, **1**:1.
15. Merry S, McDowell H, Hetrick S, Bir J, Muller N: *Cochrane Database of Systematic Reviews* 2003, **1**:1.
16. Reviews NHSCf, Dissemination: **Psychological treatment of depression in children and adolescents**. *Database of Abstracts of Reviews of Effectiveness* 2003, **1**:1.
17. Reviews NHSCf, Dissemination: **Patient and carer preference for, and satisfaction with, specialist models of palliative care**. *Database of Abstracts of Reviews of Effectiveness* 2003, **1**:1.
18. Reviews NHSCf, Dissemination: **Literature review to inform purchasing of counselling in general practice (Structured abstract)**. *Database of Abstracts of Reviews of Effectiveness* 2003, **1**:1.
19. Reviews NHSCf, Dissemination: **Do hospital-based palliative teams improve care for patients or families at the end of life? (Structured abstract)**. *Database of Abstracts of Reviews of Effectiveness* 2003, **1**:1.
20. Reviews NHSCf, Dissemination: **The effectiveness of therapeutic touch**. *Database of Abstracts of Reviews of Effectiveness* 2003, **1**:1.
21. Reviews NHSCf, Dissemination: **The impact of different models of specialist palliative care on patients' quality of life**. *Database of Abstracts of Reviews of Effectiveness* 2003, **1**:1.
22. Reviews NHSCf, Dissemination: **An integrative review and meta-analysis of therapeutic touch research (Structured abstract)**. *Database of Abstracts of Reviews of Effectiveness* 2003, **1**:1.
23. Rose S, Bisson J, Wessely S: *Cochrane Database of Systematic Reviews* 2003, **1**:1.
24. Rumbold A, Crowther CA: *Cochrane Database of Systematic Reviews* 2003, **1**:1.
25. Scott JR: *Cochrane Database of Systematic Reviews* 2003, **1**:1.

26. Stroebe M, Strobe W, Schut H: **Bereavement research: methodological issues and ethical concerns**. *Palliative Medicine* 2003, **17**(3):235-240.
27. Van P, Meleis AI: **Coping with grief after involuntary pregnancy loss: perspectives of African American women**. *JOGNN - Journal of Obstetric, Gynecologic, & Neonatal Nursing* 2003, **32**(1):28-39.

1988-89 (1)

1. Sabatini L: **Evaluating a treatment program for newly widowed people**. *Omega* 1988-89, **19**(3):229-237.
